# Associations Between Attention-Deficit/Hyperactivity Disorder and Various Eating Disorders: A Swedish Nationwide Population Study Using Multiple Genetically Informative Approaches

**DOI:** 10.1016/j.biopsych.2019.04.036

**Published:** 2019-10-15

**Authors:** Shuyang Yao, Ralf Kuja-Halkola, Joanna Martin, Yi Lu, Paul Lichtenstein, Claes Norring, Andreas Birgegård, Zeynep Yilmaz, Christopher Hübel, Hunna Watson, Jessica Baker, Catarina Almqvist, Eating Disorders Working Group of the Psychiatric Genomics Consortium, Roger Adan, Tetsuya Ando, Jessica Baker, Andrew Bergen, Wade Berrettini, Andreas Birgegård, Claudette Boni, Vesna Boraska Perica, Harry Brandt, Roland Burghardt, Matteo Cassina, Carolyn Cesta, Maurizio Clementi, Joni Coleman, Roger Cone, Philippe Courtet, Steven Crawford, Scott Crow, James Crowley, Unna Danner, Oliver Davis, Martina de Zwaan, George Dedoussis, Daniela Degortes, Janiece DeSocio, Danielle Dick, Dimitris Dikeos, Monika Dmitrzak-Weglarz, Elisa Docampo, Karin Egberts, Stefan Ehrlich, Geòrgia Escaramís, Tõnu Esko, Xavier Estivill, Angela Favaro, Fernando Fernández-Aranda, Manfred Fichter, Chris Finan, Krista Fischer, Manuel Föcker, Lenka Foretova, Monica Forzan, Christopher Franklin, Héléna Gaspar, Fragiskos Gonidakis, Philip Gorwood, Monica Gratacos, Sébastien Guillaume, Yiran Guo, Hakon Hakonarson, Katherine Halmi, Konstantinos Hatzikotoulas, Joanna Hauser, Johannes Hebebrand, Sietske Helder, Judith Hendriks, Beate Herpertz-Dahlmann, Wolfgang Herzog, Christopher Hilliard, Anke Hinney, Laura Huckins, James Hudson, Julia Huemer, Hartmut Imgart, Hidetoshi Inoko, Susana Jiménez-Murcia, Craig Johnson, Jenny Jordan, Anders Juréus, Gursharan Kalsi, Debora Kaminska, Allan Kaplan, Jaakko Kaprio, Leila Karhunen, Andreas Karwautz, Martien Kas, Walter Kaye, James Kennedy, Martin Kennedy, Anna Keski-Rahkonen, Kirsty Kiezebrink, Youl-Ri Kim, Kelly Klump, Gun Peggy Knudsen, Bobby Koeleman, Doris Koubek, Maria La Via, Mikael Landén, Robert Levitan, Dong Li, Paul Lichtenstein, Lisa Lilenfeld, Jolanta Lissowska, Pierre Magistretti, Mario Maj, Katrin Mannik, Nicholas Martin, Sara McDevitt, Peter McGuffin, Elisabeth Merl, Andres Metspalu, Ingrid Meulenbelt, Nadia Micali, James Mitchell, Karen Mitchell, Palmiero Monteleone, Alessio Maria Monteleone, Preben Mortensen, Melissa Munn-Chernoff, Benedetta Nacmias, Ida Nilsson, Claes Norring, Ioanna Ntalla, Julie O’Toole, Jacques Pantel, Hana Papezova, Richard Parker, Raquel Rabionet, Anu Raevuori, Andrzej Rajewski, Nicolas Ramoz, N. William Rayner, Ted Reichborn-Kjennerud, Valdo Ricca, Stephan Ripke, Franziska Ritschel, Marion Roberts, Alessandro Rotondo, Filip Rybakowski, Paolo Santonastaso, André Scherag, Ulrike Schmidt, Nicholas Schork, Alexandra Schosser, Jochen Seitz, Lenka Slachtova, P. Eline Slagboom, Margarita Slof-Op’t Landt, Agnieszka Slopien, Tosha Smith, Sandro Sorbi, Eric Strengman, Michael Strober, Patrick Sullivan, Jin Szatkiewicz, Neonila Szeszenia-Dabrowska, Ioanna Tachmazidou, Elena Tenconi, Laura Thornton, Alfonso Tortorella, Federica Tozzi, Janet Treasure, Artemis Tsitsika, Konstantinos Tziouvas, Annemarie van Elburg, Eric van Furth, Tracey Wade, Gudrun Wagner, Esther Walton, Hunna Watson, D. Blake Woodside, Shuyang Yao, Zeynep Yilmaz, Eleftheria Zeggini, Stephanie Zerwas, Stephan Zipfel, Lars Alfredsson, Ole Andreassen, Harald Aschauer, Jeffrey Barrett, Vladimir Bencko, Laura Carlberg, Sven Cichon, Sarah Cohen-Woods, Christian Dina, Bo Ding, Thomas Espeseth, James Floyd, Steven Gallinger, Giovanni Gambaro, Ina Giegling, Stefan Herms, Vladimir Janout, Antonio Julià, Lars Klareskog, Stephanie Le Hellard, Marion Leboyer, Astri Lundervold, Sara Marsal, Morten Mattingsdal, Marie Navratilova, Roel Ophoff, Aarno Palotie, Dalila Pinto, Samuli Ripatti, Dan Rujescu, Stephen Scherer, Laura Scott, Robert Sladek, Nicole Soranzo, Lorraine Southam, Vidar Steen, H-Erich Wichmann, Elisabeth Widen, Gerome Breen, Cynthia Bulik, Laura M. Thornton, Patrik K. Magnusson, Cynthia M. Bulik, Henrik Larsson

**Affiliations:** a Department of Medical Epidemiology and Biostatistics, Karolinska Institutet, Stockholm, Sweden; b Department of Clinical Neuroscience, Centre for Psychiatry Research, Karolinska Institutet, Stockholm, Sweden; c Stockholm Health Care Services, Stockholm County Council, Stockholm, Sweden; d Astrid Lindgren Children’s Hospital, Karolinska University Hospital, Stockholm, Sweden; e School of Medical Sciences, Örebro University, Örebro, Sweden; f MRC Centre for Neuropsychiatric Genetics and Genomics, Cardiff University, Cardiff, UK; g Social, Genetic and Developmental Psychiatry Centre, Institute of Psychiatry, Psychology and Neuroscience, King’s College London, London, UK; h Department of Psychiatry, University of North Carolina at Chapel Hill, Chapel Hill, North Carolina; i Department of Nutrition, University of North Carolina at Chapel Hill, Chapel Hill, North Carolina

**Keywords:** ADHD, Anorexia nervosa, Bulimia nervosa, Eating disorders, Genetic epidemiology, Polygenic risk score

## Abstract

**Background:**

Although attention-deficit/hyperactivity disorder (ADHD) and eating disorders (EDs) frequently co-occur, little is known about the shared etiology. In this study, we comprehensively investigated the genetic association between ADHD and various EDs, including anorexia nervosa (AN) and other EDs such as bulimia nervosa.

**Methods:**

We applied different genetically informative designs to register-based information of a Swedish nationwide population (*N* = 3,550,118). We first examined the familial coaggregation of clinically diagnosed ADHD and EDs across multiple types of relatives. We then applied quantitative genetic modeling in full-sisters and maternal half-sisters to estimate the genetic correlations between ADHD and EDs. We further tested the associations between ADHD polygenic risk scores and ED symptoms, and between AN polygenic risk scores and ADHD symptoms, in a genotyped population-based sample (*N* = 13,472).

**Results:**

Increased risk of all types of EDs was found in individuals with ADHD (any ED: odds ratio [OR] = 3.97, 95% confidence interval [CI] = 3.81, 4.14; AN: OR = 2.68, 95% CI = 2.15, 2.86; other EDs: OR = 4.66, 95% CI = 4.47, 4.87; bulimia nervosa: OR = 5.01, 95% CI = 4.63, 5.41) and their relatives compared with individuals without ADHD and their relatives. The magnitude of the associations decreased as the degree of relatedness decreased, suggesting shared familial liability between ADHD and EDs. Quantitative genetic models revealed stronger genetic correlation of ADHD with other EDs (.37, 95% CI = .31, .42) than with AN (.14, 95% CI = .05, .22). ADHD polygenic risk scores correlated positively with ED symptom measures overall and with the subscales Drive for Thinness and Body Dissatisfaction despite small effect sizes.

**Conclusions:**

We observed stronger genetic association with ADHD for non-AN EDs than for AN, highlighting specific genetic correlation beyond a general genetic factor across psychiatric disorders.

Attention-deficit/hyperactivity disorder (ADHD) and eating disorders (EDs)—including anorexia nervosa (AN) and bulimia nervosa (BN)—significantly impair the health and quality of life of the affected individuals 1, 2, 3, 4 and have been associated with elevated premature mortality 5, 6, 7, 8, 9. In both clinical and population settings, ADHD has been reported to be commonly comorbid with disordered eating behaviors 10, 11, 12, 13, 14, in particular with binge eating–related phenotypes, compared with restricting behaviors 13, 14, 15. In a Swedish adult sample (*N* = 1165) with clinically diagnosed EDs, the prevalence of ADHD symptoms was as high as 35% to 37% in BN and AN binge eating/purging subtype versus 18% in AN restricting subtype (15). ADHD symptoms during early childhood also predict binge-eating behaviors during adolescence 16, 17.

Importantly, common treatment strategies for ADHD and binge eating–related symptoms exist. For instance, a stimulant medication, lisdexamfetamine, with well-established beneficial effects on ADHD symptoms (18), has been shown to be effective in the short-term management of binge eating disorder (19) and to reduce the risk of binge eating relapse over 6 months (20). Stimulant medication is under consideration for the treatment of BN as well (15), warranting refined understanding of the shared etiology between ADHD and EDs.

The genetic liability for ADHD and EDs has been established separately by twin and family studies 21, 22, 23, 24 and genome-wide association studies (GWASs); single nucleotide polymorphism (SNP) heritability was .22 (SE = .01) for ADHD (25) and .20 (SE = .02) for AN (26), but the genetic overlap remains unclear. The available GWAS findings revealed a nonsignificant genetic correlation between ADHD and AN that was difficult to interpret owing to the low statistical power (27). GWASs for non-AN EDs are not available, and only one twin study has reported a moderate genetic correlation (.35) between self-reported non-AN EDs (i.e., binge eating behaviors) and ADHD symptoms in a Swedish adult twin sample (28). Replications at population level with clinically stringent definitions for both ADHD and disordered eating are needed.

Although shared heritability has been reported across multiple psychiatric disorders 27, 29, 30, it has not been obvious for ADHD and EDs. Moreover, various EDs might be genetically differently associated with ADHD given their differential phenotypic associations [e.g., binge eating behaviors are more correlated with ADHD compared with restricting behaviors 13, 14, 15] and warrant further investigation. We used multiple complementary approaches to comprehensively explore the phenotypic and genetic associations between ADHD and various EDs at both diagnostic and symptomatic levels. With the Swedish national register data, we first examined the familial coaggregation of clinically diagnosed ADHD and EDs, specifically AN and other EDs (OEDs), including BN. We then applied quantitative genetic modeling to estimate the genetic and environmental correlations between ADHD and EDs. Next, we incorporated GWAS findings and symptom measures in a child and adolescent twin sample, the Child and Adolescent Twin Study in Sweden (CATSS), where we derived polygenic risk scores (PRSs) for ADHD and AN separately and estimated the associations between ADHD PRSs and ED symptoms and between AN PRSs and ADHD symptoms, respectively. Convergent results across these methodologies would allow us to draw more definitive conclusions regarding the nature of the relation between ADHD and EDs.

## Methods and Materials

The use of the Swedish national registers and CATSS was approved by the Regional Ethics Review Board in Stockholm, Sweden. In the CATSS, informed consent was provided by parents (for twins aged 9 or 12 years) and by twins themselves (aged 15 years) (31).

### Data Sources

#### The Swedish National Registers

Using the unique individual identification numbers, we linked several registers in Sweden (data updated until December 2013). We acquired information on birth year, death date, and migration type and date from the Total Population Register (32); clinically diagnosed ADHD and EDs from the National Patient Register (based on the ICD-9 or ICD-10) (33), Prescribed Drug Register (34), and several treatment quality registers (based on the DSM-IV) 35, 36, 37, 38; and familial relatedness from the Multi-Generation Register. The description of these registers is detailed in the Supplement.

We identified ADHD based on registered lifetime diagnosis and medication prescriptions (23) and identified EDs (including any ED, AN, OED, and BN) based on lifetime diagnosis (9) (detailed in Supplement). Any ED, AN, and BN were defined as in previous research (9). OED was defined as having any lifetime ED other than AN (i.e., non-AN ED).

#### Child and Adolescent Twin Study in Sweden

CATSS is an ongoing study (since 2004) targeting all twins born in Sweden since July 1, 1992 (31) (data updated until December 2015). Parent reports on mental health of the twins were collected when the twins were 9 years old (born 1995–2005) or 12 years old (born 1992–1995). Follow-up questionnaires were distributed to both parents and twins when the twins reached 15 and 18 years of age. Genotype data were available for 13,472 individuals after standard data processing and quality control (Supplement).

Parent-reported ADHD symptoms were measured with a validated instrument, the Autism–Tics, ADHD, and Other Comorbidities inventory (39), at 9 or 12 years of age. The ADHD measures contained 19 items, with subscales of Inattention (9 items) and Hyperactivity/impulsivity (10 items). Questions were answered on a scale of “no” (coded 0), “yes, to some extent” (coded 0.5), and “yes” (coded 1) (31). Self-reported ED symptoms were measured by 3 of 11 subscales in the Eating Disorder Inventory-2 (EDI-2) (40), validated in Nordic countries in female individuals 41, 42, at 15 years of age, including the subscales of Drive for Thinness (7 items), Bulimia (7 items), and Body Dissatisfaction (8 items). Questions were answered on a scale with 6 options ranging from “never” (coded 1) to “always” (coded 6). We computed the sum score of the ADHD symptom questions and mean score of EDI-2 questions at both full scale and subscales (score distributions detailed in Supplemental Figure S1).

### Statistical Analysis

#### Associations Between ADHD and EDs and Familial Coaggregation

Analyses were performed in a Swedish nationwide population born in Sweden between 1970 and 2005 after excluding individuals who died or emigrated before 6 years of age and adoptees (*N* = 3,550,118; age range = 8–44 years, mean = 26.6, SD = 10.3). To study familial coaggregation, we used data for full-siblings (4,191,852 pairs), maternal half-siblings (697,763 pairs), paternal half-siblings (829,126 pairs), and cousins (16,347,002 pairs).

We first estimated odds ratios (ORs) of any ED, AN, OED, and BN in individuals with ADHD compared with individuals without ADHD using logistic regression. We then evaluated familial coaggregation patterns of ADHD and EDs by estimating ORs of EDs in each type of relative of individuals with ADHD compared with the same type of relative of individuals without ADHD. If ADHD and EDs share genetic and/or family environmental causes, ORs of EDs would be above 1 in relatives of individuals with ADHD (i.e., familial coaggregation). We adjusted for birth year and sex and addressed nonindependence of data due to familial clustering with robust (sandwich) estimator of standard errors in all regression models. We further adjusted for ADHD in the relatives as sensitivity tests 9, 43 (Supplement).

#### Quantitative Genetic Modeling

We used full-sisters and maternal half-sisters for the quantitative genetic analyses. Because the prevalence of EDs was too low in male individuals, we restricted the analyses to female individuals. We randomly selected one pair of full-sisters or maternal half-sisters from each family, resulting in 334,433 pairs of full-sisters (age range = 9–44 years, mean = 26.5, SD = 9.6) and 57,036 pairs of maternal half-sisters (age range = 9–44 years, mean = 26.2, SD = 9.3). Twin pairs were excluded because their genetic and/or environmental sharing was potentially higher than that of full-sisters who were not twins.

Each binary trait (i.e., ADHD, AN, OED, or BN) was analyzed in a liability threshold setting (44). We first estimated tetrachoric correlations, including within-trait cross-sister, phenotypic (i.e., cross-trait within-individual), and cross-trait cross-sister correlations (Supplement). Next, bivariate quantitative genetic models were fitted to quantify genetic and environmental contributions to ADHD and EDs and their associations. Analyses were performed using OpenMx (version 2.7.9) in R 3.3.2 (45). Quantitative genetic modeling decomposes variance of each disorder and covariance between two disorders into additive genetic effects (A), dominant genetic effects (D), shared environmental effects (C), and unique environmental effects (E, including measurement error). On average, full-sisters share additive (coefficient .50) and dominant (coefficient .25) genetic variance and shared (coefficient 1.00) environment variance, whereas maternal half-sisters share additive (coefficient .25) genetic variance and shared (coefficient 1.00) environment variance. For each combination of ADHD and ED, we fitted bivariate models that included A, C, and E components (ACE model), A, D, and E components (ADE model), and A and E components (AE model) and interpreted results of the best-fit model (measured by the lowest Akaike information criterion. The main results included heritability, coheritability (the proportion of the phenotypic covariance explained by the genetic covariance), and the genetic correlation for ADHD and ED.

#### Molecular Genetic Approach Using PRSs

We derived ADHD and AN PRSs for 13,472 eligible individuals in CATSS using imputed genetic data (Supplement). ADHD PRSs were generated based on independent summary statistics from the largest available GWAS of clinically diagnosed ADHD (19,099 cases and 34,194 controls, European ancestry) (46). We followed standard procedure (25) and derived ADHD PRSs based on 84,969 SNPs after linkage disequilibrium clumping (*r*^2^ > .1 within 1000 kb; with minor allele frequency ≥ 0.05 and acceptable imputation quality INFO ≥ 0.80) using 1000 genomes (European ancestry) as reference (47). AN PRSs in CATSS were derived using independent summary statistics of the largest available AN GWAS (3495 cases and 10,982 controls, European ancestry; 84,278 SNPs after linkage disequilibrium clumping with the above parameters) (26). PRSs were calculated in each individual by scoring the number of reference alleles (weighted by the allelic effect size) across the set of remaining SNPs after clumping (PLINK 1.9; http://pngu.mgh.harvard.edu/purcell/plink/) (48). We derived and standardized ADHD and AN PRSs at the *p*-value threshold *p* < 1.00 and used them for primary analyses (49); PRSs at stricter thresholds (*p* < .00001, *p* < .001, *p* < .01, *p* < .05, *p* < .10, and *p* < .50) were derived and standardized for sensitivity analyses.

We evaluated the associations between ADHD PRSs and the ED symptom score (dependent variable) and between AN PRSs and the ADHD symptom score (dependent variable) using linear regressions, adjusted for sex, birth year, and the top 5 principal components; variance in symptom score explained by PRSs was measured by the difference in *R*^2^ between the full model and the nested model without PRSs. To account for the correlated nature of the data, we applied generalized estimating equations to estimate the regression coefficients (β) and the standard error [R package drgee (50)]. β can be interpreted as the change in the symptom score per standard deviation of PRS. We stratified analyses by sex in sensitivity analyses.

## Results

The prevalence of ADHD was 3.1% in the Swedish nationwide population (*N* = 3,550,118; 2.2% in female individuals and 3.8% in male individuals) during the observed period. As shown in Table 1, individuals with ADHD had significantly higher prevalence of EDs than individuals without ADHD; the prevalence of all EDs in the study population during the observed period was higher in female individuals than in male individuals and was stable across different types of relatives (Supplemental Table S1).

**Table 1:** Descriptive Statistics of the Total Study Population and Each Type of Relative

	Total Population (*N* = 3,550,118)				Female Individuals (*n* = 1,726,311)				Male Individuals (*n* = 1,823,807)			
	ADHD		No ADHD		ADHD		No ADHD		ADHD		No ADHD	
	Number	%	Number	%	Number	%	Number	%	Number	%	Number	%
Total	108,443		3,441,675		38,339		1,687,972		70,104		1,753,703	
Any ED	2887	2.7	30,030	0.9	2588	6.8	28,260	1.7	299	0.4	1770	0.1
AN	998	0.9	14,217	0.4	916	2.4	13,425	0.8	82	0.1	792	0.0
OEDs	2586	2.4	22,962	0.7	2323	6.1	21,659	1.3	263	0.4	1303	0.1
BN	741	0.7	7090	0.2	709	1.8	6938	0.4	32	0.0	152	0.0

ADHD, attention-deficit/hyperactivity disorder; AN, anorexia nervosa; BN, bulimia nervosa; ED, eating disorder; OEDs, other eating disorders (i.e., non-AN eating disorders).

### Associations Between ADHD and EDs and Familial Coaggregation

**Figure 1:**
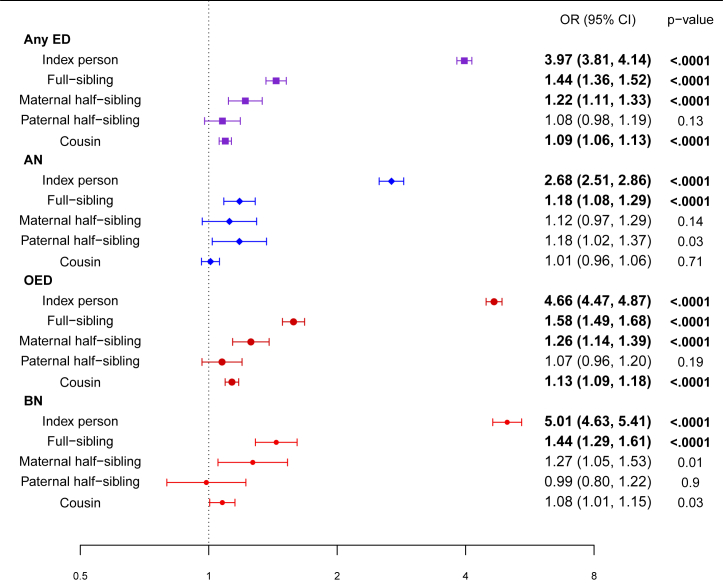
Odd ratios (ORs) of eating disorders (EDs) in individuals with attention-deficit/hyperactivity disorder (ADHD) and their relatives compared with individuals without ADHD and their relatives. The forest plot shows the ORs of any ED, anorexia nervosa (AN), other EDs (i.e., non-AN EDs) (OED), and bulimia nervosa (BN) in index individuals with ADHD and their relatives compared with index individuals without ADHD and their relatives. In general, greater ORs were found in more closely (genetically and familial environmentally) related relatives, suggesting shared genetic and/or familial environmental liabilities between ADHD and these EDs. In general, the ORs appeared to be higher for OED and BN compared with AN in each type of relative, suggesting stronger associations with ADHD in OED and BN than in AN. Bolded values are significant at *p* < .0001. CI, confidence interval.

In both sexes, individuals with ADHD had increased risk of any ED (female OR = 3.95, 95% confidence interval [CI] = 3.78, 4.12; male OR = 3.88, 95% CI = 3.41, 4.42). The ORs of OEDs (female = 4.63, 95% CI = 4.42, 4.84; male = 4.57, 95% CI = 3.98, 5.26) and BN (female = 4.94, 95% CI = 4.56, 5.35; male = 7.30, 95% CI = 4.87, 10.94) were higher than the OR of AN (female = 2.70, 95% CI = 2.52, 2.90; male = 2.29, 95% CI = 1.82, 2.89); sex difference was not statistically significant (Supplemental Table S2), with ORs in female and male individuals being comparable (Supplemental Table S3). We combined data from both sexes for familial coaggregation analyses to maximize statistical power. Full-siblings of individuals with ADHD were at increased risk of any ED (OR = 1.44, 95% CI = 1.36, 1.52), AN (OR = 1.18, 95% CI = 1.08, 1.29), OEDs (OR = 1.58, 95% CI = 1.49, 1.68), and BN (OR = 1.44, 95% CI = 1.29, 1.61) compared with full-siblings of individuals without ADHD (Figure 1). Other relatives of individuals with ADHD were also at increased risk of EDs and the magnitude of ORs was attenuated with decreasing genetic and/or familial environmental relatedness, suggesting familial liability shared between ADHD and EDs. Familial coaggregation remained statistically significant for ADHD and any ED and OED after adjusting for ADHD in relatives, further supporting the shared familial liabilities (Supplemental Table S4).

### Quantitative Genetic Modeling

The ORs of EDs in the quantitative genetic modeling sample were comparable to those in the female population (Supplemental Table S3). AE models had the lowest Akaike information criterion (Supplemental Table S5) and were therefore interpreted (Table 2).

**Table 2:** Numbers, Correlations, and Results in Quantitative Genetic Modeling for ADHD and ED (AN, OEDs, and BN)

		Number of Individuals or Pairs				Correlation			Results of Quantitative Genetic Modeling					
		Within Disorder		ED and ADHD		Within Disorder	ED and ADHD		Proportion of Variance Within Disorder Explained (95% CI)		Proportion of Covariance With ADHD Explained (95% CI)		Correlations With ADHD (95% CI)	
		Concordant Pairs^a^ (Both Affected/Both Unaffected)	Discordant Pairs^b^	With Both Disorders	Concordant Pairs^c^	Within-Trait Cross-Sister Correlation (95% CI)	Phenotypic Correlation With ADHD (95% CI)	Cross-Trait Cross-Sister Correlation With ADHD (95% CI)	Additive Genetic (A)	Unique Environmental (E)	Additive Genetic (A)	Unique Environmental (E)	Additive Genetic (A)	Unique Environmental (E)
ADHD	Full-sister	787/322,529	11,117	–	–	.41 (.39, .42)	–	–	.82 (.78, .85)	.18 (.15, .22)	–	–	–	–
	Maternal half-sister	292/52,031	4713	–	–	.22 (.19, .25)	–	–						
AN	Full-sister	107/328,436	5890	343	150	.21 (.18, .25)	.19 (.17, .21)	.04 (.04, .04)	.42 (.35, .49)	.58 (.52, .65)	.42 (.16, .69)	.58 (.31, .84)	.14 (.05, .22)	.33 (.18, .48)
	Maternal half-sister	5/56,103	928	99	44	.03 (−.10, .15)	.17 (.13, .21)	.004 (−.05, .06)						
OEDs	Full-sister	250/324,912	9271	853	331	.23 (.20, .25)	.31 (.30, .33)	.11 (.09, .13)	.44 (.39, .49)	.56 (.51, .61)	.73 (.60, .85)	.27 (.15, .40)	.37 (.31, .42)	.26 (.14, .38)
	Maternal half-sister	19/55,133	1884	286	107	.02 (−.05, .10)	.28 (.25, .31)	.04 (.01, .08)						
BN	Full-sister	34/331,325	3074	273	87	.20 (.16, .24)	.28 (.26, .30)	.07 (.07, .07)	.40 (.35, .51)	.60 (.50, .70)	.58 (.35, .81)	.42 (.19, .65)	.28 (.20, .39)	.33 (.15, .53)
	Maternal half-sister	4/56,461	571	79	37	.13 (−.02, .27)	.23 (.19, .28)	.07 (.01, .13)						

Correlations were tetrachoric correlations (presented with 95% CI). Within-trait cross-sister correlations were the tetrachoric correlations of a disorder between two sisters in a pair. Phenotypic correlations with ADHD were the tetrachoric correlations between ADHD and ED within an individual. Cross-sister cross-trait correlations with ADHD were the tetrachoric correlations between ADHD in one sister and ED in the other sister in a pair. Results are from three bivariate AE models for ADHD–AN, ADHD–OEDs, and ADHD–BN. Results are presented as point estimates (95% CI). ADHD heritability was estimated in each combination of ADHD and ED, and the estimates were similar. The presented heritability and variance explained by unique environmental variance were extracted from the bivariate AE model of ADHD–OED, which was the best powered model compared with the other two models. The heritability of ADHD was estimated to be approximately 82%, and the heritabilities of the EDs were estimated to be approximately 40% to 45%. Approximately 42% of the phenotypic covariance between ADHD and AN was explained by their genetic covariance, whereas approximately 73% of the phenotypic covariance between ADHD and OEDs was explained by their genetic covariance, and the proportion for ADHD and BN was estimated to be approximately 58%. The remaining proportion of the phenotypic covariance between ADHD and each ED was explained by their unique environmental covariance. The genetic correlation between ADHD and AN was estimated to be approximately .14 (.05, .22), whereas higher genetic correlation with ADHD was found in OEDs (.37 [.31, .42]) and potentially also in BN (.28 [.20, .39]).

ADHD, attention-deficit/hyperactivity disorder; AN, anorexia nervosa; BN, bulimia nervosa; CI, confidence interval; ED, eating disorder; OEDs, other eating disorders (i.e., non-AN eating disorders).

a The number of pairs with both sisters affected vs. (/) the number of pairs with both sisters unaffected.

b The number of pairs where one sister was affected with the disorder and the other was unaffected.

c The number of pairs where one sister was affected with ADHD and the other affected with ED.

As shown in Table 2, within-trait cross-sister correlations for ADHD, AN, OEDs, and BN were higher in full-sisters than in maternal half-sisters, suggesting genetic effects on each disorder given that the two types of sisters were assumed to have equal environmental sharing but that full-sisters share more genetic variance than maternal half-sisters. Heritability was estimated to be 82% in ADHD, 42% in AN, 45% in OEDs, and 40% in BN from the AE models.

The phenotypic correlation between ADHD and each ED was comparable between sister types; OEDs manifested higher phenotypic correlation with ADHD than AN, which agreed with the observed pattern of ORs of OEDs and AN in individuals with and without ADHD. The cross-trait cross-sister correlations between ADHD and EDs were also higher in full-sisters than in maternal half-sisters, suggesting genetic effects on the association between ADHD and each ED. The coheritability with ADHD was estimated to be 73% for OEDs, suggesting that 73% of the phenotypic covariance between OEDs and ADHD was explained by genetic covariance of the two disorders. Approximately 58% of the phenotypic covariance between BN and ADHD and 42% of the phenotypic covariance between AN and ADHD were attributable to their genetic covariance. Genetic correlation with ADHD was numerically higher in OEDs (.37, 95% CI = .31, .42), and potentially in BN (.28, 95% CI = .20, .39), than in AN (.14, 95% CI = .05, .22).

### PRS Analysis

The subscales of ADHD and ED symptoms showed high internal consistency except the subscale Bulimia [Cronbach’s alpha (51)] (Table 3). ADHD PRSs were significantly associated with the EDI-2 full scale (β = .027, 95% CI = .005, .049, *R*^2^ = .0012, *p* = .015) and the subscales Drive for Thinness (β = .032, 95% CI = .005, .059, *R*^2^ = .0010, *p* = .022) and Body Dissatisfaction (β = .042, 95% CI = .011, .072, *R*^2^ = .0013, *p* = .007) but not the subscale Bulimia (β = .004, 95% CI = −.013, .021, *p* = .654) (Table 3). In contrast, AN PRSs were not significantly associated with ADHD full scale or the subscales.

**Table 3:** Associations Between ADHD PRS and ED Symptom Measures and Between AN PRS and ADHD Symptom Measures

	Individual^a^, *n* (%)	Symptom Measures, Mean (SD)	Cronbach’s Alpha	Regression Coefficient, β (95% CI)	*p* Value	*R*^2^
ADHD PRS and ED Symptom Measures						
EDI-2 full scale (range: 1–5.8)	5680 (42.2)	2.1 (0.77)	.92	.027 (.005, .049)	.015	.0012
Drive for Thinness (range: 1–6)	5674 (42.1)	2.1 (0.98)	.89	.032 (.005, .059)	.022	.0010
Bulimia (range: 1–6)	5668 (42.1)	1.5 (0.57)	.72	.004 (−.013, .021)	.654	.0000
Body Dissatisfaction (range: 1–6)	5679 (42.2)	2.6 (1.13)	.90	.042 (.011, .072)	.007	.0013
AN PRS and ADHD Symptom Measures						
ADHD full scale (range: 0–19)	13,451 (99.8)	1.8 (2.89)	.96	−.049 (−.101, .002)	.062	.0003
Inattention (range: 0–9)	13,454 (99.9)	1.0 (1.65)	.94	−.029 (−.058, .000)	.053	.0003
Impulsivity/hyperactivity (range: 0–10)	13,455 (99.9)	0.9 (1.57)	.93	−.021 (−.049, .007)	.145	.0002

The table shows the results of the primary analysis, where ADHD PRS and AN PRS were derived based on all single nucleotide polymorphisms after linkage disequilibrium clumping (*p*-value threshold < 1) and standardized before analysis. ADHD PRSs were significantly associated with symptom measures of Drive for Thinness (*p* = .022), Body Dissatisfaction (*p* = .007), and the full scale (*p* = .015), but they were not significantly associated with the measure of Bulimia. *R*^2^ represents the proportion of variance in the symptom measures explained by the variance in the PRS; for example, the variance in ADHD PRS explained approximately 0.1% variance in the measure of Drive for Thinness (*R*^2^ = .0010) and approximately 0.13% variance in the measure of Body Dissatisfaction (*R*^2^ = .0013). Regression coefficient (β) reflects the change in symptom measures per standard deviation increase of the PRS; for example, when ADHD PRS increased by 1 standard deviation, the symptom measure would increase by 0.032 points for Drive for Thinness, by 0.042 for Body Dissatisfaction, and by 0.027 for the full scale. AN PRS was not significantly associated with any of the symptom measures of ADHD. Standardized Cronbach’s alpha was presented as a measure for internal consistency within each (sub)scale; higher values correspond to higher internal consistency. The range for acceptable values was .70 to .95.

ADHD, attention-deficit/hyperactivity disorder; AN, anorexia nervosa; CI, confidence interval; ED, eating disorder; EDI-2, Eating Disorder Inventory-2; PRSs, polygenic risk scores.

a The total number (%) of individuals with each symptom measure in the study population (*N* = 13,472) is shown.

**Figure 2:**
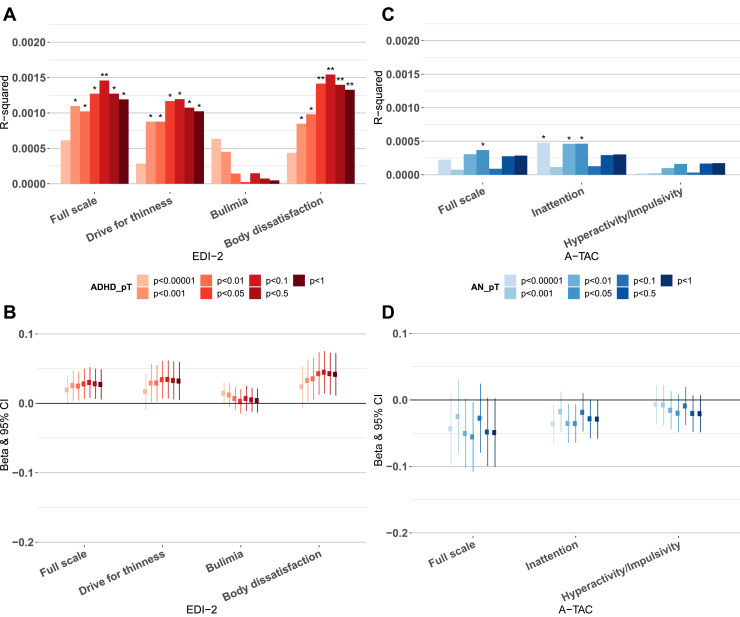
Variance explained (*R*^2^) and regression coefficient (β) for the association between attention-deficit/hyperactivity disorder (ADHD) polygenic risk scores (PRSs) and eating disorder (ED) symptoms and the association between anorexia nervosa (AN) PRSs and ADHD symptoms. **(A, B)** Associations between ADHD PRSs and Eating Disorder Inventory-2 (EDI-2) measures of ED symptoms. Panel **(A)** shows *R*^2^, and panel **(B)** shows β and 95% confidence interval (CI). ADHD PRSs across different *p*-value thresholds showed consistent results in explaining the variance **(A)** and consistent regression coefficients **(B)** in each measure of the ED symptoms. **(C, D)** Associations between AN PRSs and Autism–Tics, ADHD, and Other Comorbidities inventory (A-TAC) measures for ADHD symptoms. Panel **(C)** shows *R*^2^, and panel **(D)** shows β and 95% CI. Variance explained **(C)** and regression coefficients **(D)** were less consistent for the associations between AN PRSs and ADHD. AN PRSs across multiple *p*-value thresholds were not significantly associated with measures of ADHD symptoms in general, although AN PRSs at *p*-value thresholds *p* < .00001, *p* < .01, and *p* < .05 showed negative associations with the measure of Inattention (*p* < .05). ADHD_pT, *p*-value thresholds for ADHD PRSs; AN_pT, *p*-value thresholds for AN PRSs. PRSs at threshold *p* < 1.00 were used for the main analysis. PRSs at other *p*-value thresholds were used for sensitivity tests. *p ≤ .05 and **p ≤ .01 for the associations between PRSs and the symptom measures.

The sensitivity analyses of ADHD PRSs at different *p*-value thresholds showed consistency in variance explained (Figure 2A) and regression coefficient (Figure 2B) for all EDI-2 measures; the associations appeared to be stronger in female individuals, but sex differences were not statistically significant (Supplemental Table S7). The associations between AN PRSs and ADHD symptoms were less consistent. AN PRSs at the *p*-value thresholds *p* < .00001, *p* < .01, and *p* < .05 showed weak negative associations with the subscale Inattention (Figure 2C, D), which might be driven by male individuals who had higher variation in the symptoms than female individuals (Supplemental Tables S8 and S9 and Supplemental Figures S2 and S3).

## Discussion

Although genetic correlations are found across several psychiatric disorders 27, 30, little is known about how various EDs genetically correlate with other disorders, specifically ADHD, given their common clinical co-occurrence 11, 12, 15, 16. Here we reported convergent results from different genetically informative study designs revealing the genetic associations with ADHD across various EDs, especially non-AN EDs compared with AN, highlighting specific genetic correlations beyond a general genetic factor across psychiatric disorders (29). Our finding of marked genetic correlations between ADHD and non-AN EDs was in line with a previously found genetic correlation between ADHD symptoms and binge eating behaviors (28) and might in part explain the stronger associations of ADHD with binge eating–related symptoms than with restrictive symptoms 13, 14, 15, 17. We further found that common genetic risk variants for ADHD were significantly associated with ED symptoms, which was also consistent with our familial coaggregation and quantitative genetic findings. Our results enhance the understanding of why EDs and ADHD co-occur and underscore the importance of screening for comorbid symptoms in individuals with EDs and ADHD.

To explore the genetic overlap, we used 3 analytical approaches: familial coaggregation analysis, quantitative genetic modeling, and a molecular genetic approach using PRSs. Converging evidence across different methods strengthens the support for a genetic overlap between ADHD and EDs. It also demonstrates that the genetic associations with ADHD are present not only in clinically diagnosed EDs (which may represent the extreme end of a symptom spectrum) but also in dimensional ED traits in the general population. The findings complement discussions about the categorical versus dimensional conceptualizations of mental disorders 30, 52 and show the value in using both types of measures in genomic research.

Our findings have important clinical implications. First, the observed associations and familial coaggregation patterns between ADHD and EDs suggest that family history may help with early detection and risk identification of the two disorders. ADHD has been shown to predict ED symptoms (16); regular screening for ADHD symptoms in those with EDs and for ED symptoms in those with ADHD may hasten detection of and appropriate intervention for comorbid conditions. When interpreting the estimated ORs, it is important to acknowledge that other comorbidities were not accounted for because the adjustment could introduce bias (53) owing to the complex yet unclear interdependencies between disorders. Second, our findings marked stronger phenotypic and genetic correlations with ADHD in non-AN EDs compared with AN, implying different etiologies and potential treatment strategies among patients with ADHD with different comorbid ED conditions. Stimulant medication has been shown to be effective in both ADHD (18) and binge eating disorder 19, 20, and applying stimulant medication to treat BN is under consideration (15). Our finding of shared genetic underpinnings of ADHD and non-AN EDs may provide further support for common treatment strategies for the two disorders. Third, recent studies have reported an association between ADHD and obesity 54, 55, 56, and disordered eating has been hypothesized to affect this association 57, 58. Shared etiology of ADHD and disordered eating implied by our study may support this hypothesis and encourages more detailed investigation to determine the extent to which disordered eating contributes to obesity in individuals with ADHD.

The genetic overlap between ADHD and EDs may in part reflect a more general genetic susceptibility to psychopathology 29, 59. Our finding that the genetic sharing of ADHD was stronger with non-AN EDs than with AN does, nevertheless, support genetic specificity beyond such a general genetic factor. For instance, genetic factors underlying (food-related) impulsivity (60) might be of specific importance for the observed genetic overlap between ADHD and non-AN EDs. How BN PRSs correlate with different subscales of ADHD will be of value once BN GWASs become available.

Despite the fact that PRSs were statistically significantly associated with the phenotype measures, their effect sizes were generally small, possibly owing to 1) PRSs being based on tagged but not necessarily causal SNPs, 2) polygenic measures possibly not reflecting more complex genetic architecture (61), and 3) the effect of each genetic variant being estimated with error, which may be improved with increased discovery sample size [as seen in schizophrenia (62)]. These may also explain the discrepancy between our results of positive ADHD–AN genetic correlation and the nonsignificant association between AN PRSs and ADHD symptoms, which was nevertheless consistent with previous genomic findings (27). Increased proportion of phenotypic variance explained by PRSs is anticipated with increased discovery sample sizes and PRSs that better capture causal genetic effects.

Limitations need to be considered. First, register-based data captured only treatment-seeking individuals. Individuals who had EDs but did not seek treatment were therefore misclassified in our study. BN was not identifiable in the Swedish version of ICD-9, resulting in additional misclassification. If the misclassification of EDs was differential for ADHD diagnosis (e.g., the diagnosis of ADHD increased the chance for discovering EDs in the individual) and vice versa, the associations between ADHD and EDs might be overestimated. Nevertheless, the diagnosis of ADHD was less likely to influence the chance of discovering EDs in a relative, so the estimated familial coaggregation might be less biased. Second, the EDI-2 subscale Bulimia had low internal consistency and low variation, which might be due to the fact that participants in the CATSS (15 years old) might have not yet developed pathological binge eating behaviors that typically begin during late adolescence or early adulthood 35, 63. Third, the study was underpowered to perform the quantitative genetic modeling in male individuals. Better detection of EDs and/or continuous symptom measures in male individuals might provide sufficient statistical power for the analyses in male individuals and examination of sex differences.

In conclusion, using 3 convergent methodological approaches, we observed significant genetic associations between clinically diagnosed ADHD and EDs as well as dimensional measures of ED pathology. The genetic association with ADHD was stronger in non-AN EDs compared with AN, highlighting specific genetic correlations beyond a general genetic factor across psychiatric disorders. We expect that as genetic sample sizes grow, PRSs will become more robust and the next iteration of PRS analyses will have greater statistical power. Nonetheless, our convergent results encourage clinical vigilance for copresentation of EDs (especially non-AN EDs) and ADHD and suggest that, at least in part, the observed association between these 2 presentations is due to shared genetic factors.

## Supplementary Material

Supplemental Material

Key Resources Table
