# Supplementary material for: Associations Between Attention-Deficit/Hyperactivity Disorder and Various Eating Disorders: A Swedish Nationwide Population Study Using Multiple Genetically Informative Approaches
Source: Biol Psychiatry. 2019 Oct 15;86(8):577–86. doi: 10.1016/j.biopsych.2019.04.036 (PMC6776821; doi:10.1016/j.biopsych.2019.04.036)
Supplement: Supplemental Material [file mmc1.pdf]

Associations Between Attention-Deficit/Hyperactivity Disorder and Various Eating Disorders: A Swedish Nationwide Population Study Using Multiple Genetically Informative Approaches

Supplemental Information

Supplementary Methods

The Swedish National Registers

Data from multiple Swedish registers were de-identified and merged by an independent Swedish governmental agency (Statistics Sweden) for research purposes (1).

We acquired information on birth year and month, death date, and migration type and date from the Swedish Total Population Register (1). The National Patient Register provides clinical diagnoses based on the Swedish versions of the International Classification of Diseases, Eighth Revision (ICD-8, 1973-1986), ICD-9 (1987-1996), and ICD-10 (since 1997); it includes inpatient psychiatric diagnoses from 1973 and outpatient psychiatric diagnoses from 2001 (2). Two treatment quality registers for EDs—the Swedish National Quality Register for Eating Disorder Treatment (Riksät, since 1999) and quality assurance system for eating disorders (Stepwise, since 2005)—also provided information on clinical diagnosis of EDs with increasing coverage over time (3). Diagnoses in these two treatment quality registers were collected from specialized treatment centers across Sweden (4, 5) and were based on the Diagnostic and Statistical Manual of Mental Disorders, 4th Edition, Text Revision (DSM-IV-TR) (6). The Prescribed Drug Register (since 2005) provided information on prescribed medications for the entire population with active ingredients coded according to the anatomical therapeutic chemical classification system (7). The Clinical Database for Child and Adolescent Psychiatry in Stockholm (Pastill, since 2001) contains diagnoses of psychiatric disorders based on ICD-10 or DSM-IV from Child and Adolescent Mental Health Services in Stockholm

County (8). The Multi-Generation Register (Statistics Sweden) contains information on biological parents of individuals who were born after 1932 and lived in Sweden any time after 1961.

Diagnoses of ADHD and EDs in Registers

Clinically diagnosed ADHD was identified with ICD codes 314 (Swedish ICD-9) or F90 (ICD-10) from NPR, or F90 (ICD-10) or 314 (DSM-IV) from a treatment quality register (Pastill). We also identified ADHD from drug prescription of methylphenidate (N06BA04), amphetamine (N06BA01), dexamphetamine (N06BA02), or atomoxetine (N06BA09) from the Prescribed Drug Register.

Any ED was defined as having a diagnosis of any ED, identified with codes 307B or 307F (Swedish ICD-9) or F50.0-F50.3, or F50.9 (ICD-10) from NPR, or meeting DSM-IV criteria for EDs from the two treatment quality registers. AN included diagnoses of AN or atypical AN, identified with 307B (Swedish ICD-9) or F50.0 or F50.1 (ICD-10) from NPR, or meeting DSM-IV criteria for AN or atypical AN from the two treatment quality registers. BN included diagnosed BN or atypical BN, identified with F50.2 or F50.3 (ICD-10) from NPR, or meeting DSM-IV criteria for BN or atypical BN from the two treatment quality registers. As BN was not available in the Swedish ICD-9 and that we were unable to further distinguish BED from EDNOS in ICD-10, we also analyzed a heterogeneously defined group, other EDs (OED), defined as having a diagnosis of any ED that was not AN; individuals could have both AN and OED at different time points, and BN was a subset of OED. We were unable to distinguish between restrictive and binge-purge subtypes of AN or various subtypes of Eating Disorders Not Otherwise Specified (including binge eating disorder) from ICD codes.

Genotype Data in CATSS and Quality Control

Using the Illumina Infinium PsychArray-24 BeadChip, 11,551 CATSS participants were genotyped, among whom 11,081 individuals with 561,187 genotyped single nucleotide polymorphism loci (SNPs) passed stringent quality control (detailed processing has been described elsewhere (9)). We imputed genotypes of 2,495 monozygotic twins using their genotyped co-twins, resulting in 13,576 individuals with genotypes. Imputation on autosomes was performed in Minimac3 (10) with 1000-Genomes data (Phase 3, Version.5) (11) as the reference panel. We derived principal components (PCs) to account for population stratification in the CATSS sample using PC analysis in PLINK after LD-pruning and removing genotyped SNPs located in long-range LD regions (9). PCs were calculated in unrelated individuals and projected onto relatives. In line with previous research (9), we excluded individuals with cerebral palsy, brain injury, Down syndrome, and chromosomal abnormalities; this resulted in 13,472 individuals eligible for PRS analysis.

Deriving Polygenic Risk Score (PRS) for ADHD and AN in CATSS Genotyped Sample

We derived PRS for ADHD and AN as measures of the genetic risks of the two disorders respectively for each of the 13,472 eligible individuals in CATSS using imputed genetic data (as described above). ADHD PRS were generated based on summary statistics from the most updated GWAS of clinically diagnosed ADHD (19,099 cases and 34,194 controls restricted to European-ancestry) by The Lundbeck Foundation Initiative for Integrative Psychiatric Research (iPSYCH) and the Psychiatric Genomics Consortium (PGC) ADHD group (12); the discovery sample was independent from CATSS. We derived ADHD PRS using the same method and parameters as a recent study (9). Multi-allelic and ambiguous SNPs were excluded. Autosomal SNPs with a minor allele frequency (MAF) >= 0.05 and acceptable imputation quality (INFO>= 0.8) were clumped within 1000 kb to account for long-range linkage

disequilibrium (LD; r-squared > 0.1). This was preformed in PLINK.v.1.9 (13) and resulted in 84,969 SNPs. Based on standard procedure, we weighted each reference allele by the beta and summed the values for the set of clumped SNPs to derive ADHD PRS for each individual in CATSS (14). The same procedure was applied to derive AN PRS based on summary statistics of the largest AN GWAS to date (15). The study was performed on 3,495 AN cases and 10,982 controls; the discovery sample was independent from CATSS. After clumping with the same parameters as were used for ADHD, 84,278 SNPs remained, and AN PRS were derived using the same method as was used for ADHD. For both ADHD and AN, we derived PRS across seven p-value thresholds (p<0.00001, p<0.001, p<0.01, p<0.05, p<0.1, p<0.5, and p<1). We used threshold p<1, which contains all available SNPs after LD clumping, as the primary PRS for both ADHD and AN in main analysis (16).

Association and Familial Co-aggregation of ADHD and EDs—Sensitivity Analysis

In each of the five relative cohorts, we repeated the main familial co-aggregation analysis, i.e., regressed the relative's ED status on the index individual's ADHD status, adjusting for covariates mentioned in the main text, and adjusted for ADHD in the relative. These models were over-adjusted and provided conservative estimates. If the ORs remained significant after adjustment, the contribution of common familial liabilities to ADHD and ED would be further supported. The rationale has been clearly explained in earlier research (17). Results of this sensitivity analysis were presented in supplementary Table S3.

Correlations in Quantitative Genetic Modeling

Before variance decomposition, we first estimated correlations. They are tetrachoric correlations as the traits are binary. Within-trait cross-sister correlation (correlation of a disorder, i.e., ADHD, AN, OED, and BN, between two sisters in a pair) described similarity in

the disorder between two sisters in a pair and could imply genetic liability for the disorder if estimated higher in full-sisters than in maternal half-sisters. Phenotypic correlation (correlation of two disorders in an individual) described the overlap between ADHD and EDs and was expected to be similar between full- and half-sisters. Cross-trait cross-sister correlation (correlation of one disorder in one sister and the other disorder in the other sister in a pair) describes the overlap between ADHD and each ED in a pair and could imply genetic liability to the overlap if estimated higher in full-sisters than in maternal half-sisters. These correlations described the relationships between disorders within and across different types of relatedness, and could imply genetic and environmental effects.

Supplementary Tables and Figures

Table S1. Descriptive information of each type of relative

Full-siblings												
	Total	2,718,181			Female	1,321,209			Male	1,396,972		
	ADHD		No ADHD		ADHD		No ADHD		ADHD		No ADHD	
	No.	%	No.	%	No.	%	No.	%	No.	%	No.	%
Total	75,339		2,642,842		26,670		1,294,539		48,669		1,348,303	
Any ED	2,136	2.8%	24,416	0.9%	1,919	7.2%	22,993	1.8%	217	0.4%	1,423	0.1%
AN	755	1.0%	11,764	0.4%	692	2.6%	11,129	0.9%	63	0.1%	635	0.0%
OED	1,901	2.5%	18,567	0.7%	1,710	6.4%	17,524	1.4%	191	0.4%	1,043	0.1%
BN	542	0.7%	5,721	0.2%	516	1.9%	5,601	0.4%	26	0.1%	120	0.0%
Maternal half-siblings												
	Total	472,204			Female	230,175			Male	242,029		
	ADHD		No ADHD		ADHD		No ADHD		ADHD		No ADHD	
	No.	%	No.	%	No.	%	No.	%	No.	%	No.	%
Total	28,927		443,277		10,514		219,661		18,413		223,616	
Any ED	704	2.4%	4,520	1.0%	630	6.0%	4,237	1.9%	74	0.4%	283	0.1%
AN	222	0.8%	1,919	0.4%	205	1.9%	1,816	0.8%	17	0.1%	103	0.0%
OED	637	2.2%	3,638	0.8%	571	5.4%	3,404	1.5%	66	0.4%	234	0.1%
BN	153	0.5%	1,088	0.2%	148	1.4%	1,057	0.5%	5	0.0%	31	0.0%
Paternal half-siblings												
	Total	506,950			Female	247,847			Male	259,103		
	ADHD		No ADHD		ADHD		No ADHD		ADHD		No ADHD	
	No.	%	No.	%	No.	%	No.	%	No.	%	No.	%
Total	27,270		479,680		9,940		237,907		17,330		241,773	

Any ED	704	2.6%	4,830	1.0%	626	6.3%	4,527	1.9%	78	0.5%	303	0.1%
AN	227	0.8%	2,059	0.4%	211	2.1%	1,923	0.8%	16	0.1%	136	0.1%
OED	641	2.4%	3,847	0.8%	572	5.8%	3,627	1.5%	69	0.4%	220	0.1%
BN	160	0.6%	1,209	0.3%	154	1.5%	1,180	0.5%	6	0.0%	29	0.0%
Cousins												
	Total	2,859,445			Female	1,390,084			Male	1,469,361		
	ADHD		No ADHD		ADHD		No ADHD		ADHD		No ADHD	
	No.	%	No.	%	No.	%	No.	%	No.	%	No.	%
Total	86,078		2,773,367		30,740		1,359,344		55,338		1,414,023	
Any ED	2,380	2.8%	25,402	0.9%	2,129	6.9%	23,928	1.8%	251	0.5%	1,474	0.1%
AN	840	1.0%	12,199	0.4%	774	2.5%	11,532	0.8%	66	0.1%	667	0.0%
OED	2,143	2.5%	19,349	0.7%	1,916	6.2%	18,270	1.3%	227	0.4%	1,079	0.1%
BN	596	0.7%	5,947	0.2%	568	1.8%	5,818	0.4%	28	0.1%	129	0.0%

Note: ADHD: attention-deficit hyperactivity/impulsivity disorder, ED: eating disorder, AN: anorexia nervosa, OED: other eating disorder, i.e., non-AN eating disorders, BN: bulimia nervosa. Each type of relative was a subset of the entire study population. The prevalence of the disorders were stable across different types of relatives.

Table S2. Test for modification effect of sex on the associations between ADHD and EDs, p-value for interaction term between sex and ADHD.

	p-value
Any ED	0.812
AN	0.182
OED	0.878
BN	0.064

Note: ADHD: attention-deficit hyperactivity/impulsivity disorder, ED: eating disorder, AN: anorexia nervosa, OED: other eating disorder, i.e., non-AN eating disorders, BN: bulimia nervosa.

To test the modification effect of sex on the association between ADHD and EDs in the index individuals, an interaction term between sex and each covariate in the model was added; the p-value was estimated for the interaction term between sex and ADHD. All models were adjusted for non-independence between observations due to familial clusters with robust (sandwich) estimator of standard errors.

Table S3. OR of EDs among individuals with ADHD and their relatives, compared to individuals without ADHD and their relatives, in females and males in the population and in the randomly selected sample for quantitative genetic modelling.

		Female		Male		Quantitative genetic sample	
		OR	p-value	OR	p-value	OR	p-value
Any ED	Index person	3.95 (3.78, 4.12)	<.0001	3.88 (3.42, 4.41)	<.0001	3.92 (3.60, 4.27)	<.0001
	Full-sibling	1.42 (1.35, 1.51)	<.0001	1.62 (1.33, 1.97)	<.0001	1.52 (1.37, 1.70)	<.0001
	Maternal half-sibling	1.24 (1.13, 1.36)	<.0001	0.93 (0.63, 1.39)	0.73	1.36 (1.15, 1.62)	<.0001
	Paternal half-sibling	1.09 (0.99, 1.21)	0.08	0.85 (0.58, 1.24)	0.40	1.11 (0.91, 1.37)	0.30
	Cousin	1.09 (1.06, 1.13)	<.0001	1.09 (0.96, 1.23)	0.19	1.05 (0.98, 1.12)	0.15
AN	Index person	2.70 (2.52, 2.90)	<.0001	2.29 (1.82, 2.89)	<.0001	2.64 (2.30, 3.04)	<.0001
	Full-sibling	1.18 (1.08, 1.29)	<.0001	1.15 (0.82, 1.62)	0.42	1.26 (1.07, 1.49)	0.01
	Maternal half-sibling	1.13 (0.97, 1.31)	0.12	0.96 (0.50, 1.84)	0.90	1.22 (0.92, 1.61)	0.16
	Paternal half-sibling	1.19 (1.02, 1.39)	0.02	0.99 (0.55, 1.79)	0.99	1.38 (1.01, 1.89)	0.04
	Cousin	1.01 (0.96, 1.06)	0.63	0.96 (0.79, 1.17)	0.71	0.93 (0.84, 1.02)	0.14
OED	Index person	4.63 (4.42, 4.84)	<.0001	4.57 (3.98, 5.26)	<.0001	4.50 (4.11, 4.93)	<.0001
	Full-sibling	1.57 (1.47, 1.67)	<.0001	1.74 (1.40, 2.16)	<.0001	1.65 (1.46, 1.85)	<.0001
	Maternal half-sibling	1.28 (1.16, 1.42)	<.0001	0.92 (0.60, 1.41)	0.70	1.37 (1.14, 1.65)	0.00
	Paternal half-sibling	1.09 (0.97, 1.21)	0.15	0.91 (0.61, 1.36)	0.65	1.05 (0.84, 1.33)	0.66
	Cousin	1.13 (1.09, 1.18)	<.0001	1.13 (0.99, 1.30)	0.08	1.09 (1.01, 1.17)	0.02
BN	Index person	4.94 (4.56, 5.35)	<.0001	7.30 (4.87, 10.94)	<.0001	5.07 (4.32, 5.95)	<.0001
	Full-sibling	1.44 (1.28, 1.61)	<.0001	1.49 (0.70, 3.17)	0.31	1.47 (1.17, 1.84)	0.00
	Maternal half-sibling	1.28 (1.06, 1.55)	0.01	0.93 (0.30, 2.88)	0.91	1.47 (1.04, 2.06)	0.03
	Paternal half-sibling	1.00 (0.81, 1.24)	0.99	0.55 (0.14, 2.13)	0.39	0.69 (0.42, 1.15)	0.16
	Cousin	1.07 (1.00, 1.15)	0.05	1.27 (0.89, 1.83)	0.19	1.01 (0.88, 1.16)	0.90

Note: ADHD: attention-deficit hyperactivity/impulsivity disorder, ED: eating disorders, AN: anorexia nervosa, OED: other eating disorders, i.e., non-AN eating disorders, BN: bulimia nervosa.

The table shows OR of different EDs in index individuals with ADHD and their relatives compared to index individuals without ADHD and their relatives, in females and males in the population and in individuals in the random sample for quantitative genetic modelling, which consisted of full-sisters and maternal half-sisters. The ORs in all three groups were comparable to those in the main analysis (Figure 1).

Table S4. OR of EDs among relatives of individuals with ADHD compared to relatives of individuals without ADHD, adjusted for ADHD in the relative.

	OR (95% CI)	p
**Any ED**		
Full-siblings	1.13 (1.06, 1.19)	<.0001
Maternal half-siblings	1.07 (0.97, 1.17)	0.161
Paternal half- siblings	0.99 (0.90, 1.09)	0.866
Cousin	1.04 (1.00, 1.07)	0.03
**AN**		
Full-siblings	1.01 (0.92, 1.10)	0.901
Maternal half-siblings	1.03 (0.89, 1.20)	0.654
Paternal half-siblings	1.12 (0.96, 1.29)	0.139
Cousin	0.98 (0.93, 1.02)	0.333
**OED**		
Full-siblings	1.19 (1.12, 1.27)	<.0001
Maternal half-siblings	1.08 (0.98, 1.19)	0.139
Paternal half-siblings	0.98 (0.87, 1.09)	0.648
Cousin	1.06 (1.02, 1.10)	0.001
**BN**		
Full-siblings	1.10 (0.98, 1.24)	0.107
Maternal half-siblings	1.11 (0.92, 1.35)	0.262
Paternal half-siblings	0.92 (0.74, 1.13)	0.419
Cousin	1.02 (0.95, 1.09)	0.598

Note: ADHD: attention-deficit hyperactivity/impulsivity disorder, EDs: eating disorders, AN: anorexia nervosa, OED: other eating disorders, i.e., non-AN eating disorders, BN: bulimia nervosa, OR: odds ratio, 95% CI: 95% confidence interval. The models adjusted for ADHD in the relatives were over-adjusted models and provided conservative estimates. The OR was still significant in full-siblings (and cousins) for any ED and OED, providing an additional proof for the familial liabilities between ADHD and any ED and OED.

Table S5. Model fitting statistics compared between ACE, ADE, and AE models.

Model	-2 Log Likelihood	Degree of freedom	AIC	p-value compared to saturated model	p-value compared to AE model
ADHD-AN					
ACE	245456.7	1565863	-2886269	0.060682	0.801252
ADE	245455.9	1565863	-2886270	0.078644	0.6149349
AE	245457.7	1565866	-2886274	0.139747	.
ADHD-OED					
ACE	284962.4	1565863	-2846764	0.002575	0.7770741
ADE	284963.1	1565863	-2846763	0.001898	0.9402425
AE	284963.5	1565866	-2846768	0.008683	.
ADHD-BN					
ACE	211752.8	1565863	-2919973	0.007614	0.5519128
ADE	211756.9	1565863	-2919969	0.001641	1
AE	211754.9	1565866	-2919977	0.017213	.

Note: ADHD: attention-deficit hyperactivity/impulsivity disorder, AN: anorexia nervosa, OED: other eating disorders, i.e., non-AN eating disorders, BN: bulimia nervosa. In each combination of ADHD and EDs, ACE, ADE, and AE models were compared to the saturated models where all thresholds and covariance were free to be estimated. Likelihood ratio tests between ACE, ADE, and AE models and the saturated model and between AE models and ACE and ADE models showed that AE models fitted the data equally well as ACE and ADE models. AE models had the lowest AIC in all combinations of ADHD and EDs, suggesting that they were the most parsimonious models. Results of AE models were therefore selected for interpretation in the main text.

Note that the ADE model had worse likelihood than AE model for ADHD-BN combination due to optimization issues, which also led to failure in estimating some of the standard errors of the estimates. We additionally fitted the models using weighted least square and obtained very close estimations with standard errors. Results are presented in Table S6.

Table S6. Model fitting statistics compared between ACE, ADE, and AE models using weighted least square method

Model	-2 Log Likelihood	Degree of freedom	AIC	p-value compared to saturated model	p-value compared to AE model	A		C		D		E	
ADHD-AN						ADHD	AN	ADHD	AN	ADHD	AN	ADHD	AN
ACE	404.1732	30	343.0906	0.1318709	0.8659281	0.76 (0.62, 0.91)	0.43 (0, 0.93)	0.03 (0, 0.09)	0 (0, 0.24)	.	.	0.21 (0.13, 0.29)	0.57 (0.31, 0.83)
ADE	403.8946	30	343.0906	0.1581534	0.7989816	0.82 (0.69, 0.95)	0.19 (0, 0.67)	.	.	0.01 (0, 0.27)	0.49 (0, 1)	0.17 (0.03, 0.32)	0.32 (0, 0.82)
AE	404.904	33	338.904	0.5576285	-	0.82 (0.79, 0.86)	0.43 (0.36, 0.50)	.	.	.	.	0.18 (0.14, 0.21)	0.57 (0.50, 0.64)
ADHD-OED						ADHD	OED	ADHD	OED	ADHD	OED	ADHD	OED
ACE	419.0874	30	353.9168	0.00535216	0.8717061	0.76 (0.62, 0.91)	0.45 (0.13, 0.76)	0.03 (0, 0.09)	0 (0, 0.15)	.	.	0.21 (0.13, 0.29)	0.55 (0.38, 0.72)
ADE	414.6695	30	353.9168	0.06767762	0.1629163	0.82 (0.69, 0.95)	0.11 (0, 0.41)	.	.	0.01 (0, 0.27)	0.69 (0.09, 1)	0.17 (0.03, 0.32)	0.19 (0, 0.51)
AE	419.7938	33	353.7938	0.07602301	-	0.82 (0.79, 0.86)	0.45 (0.40, 0.50)	.	.	.	.	0.18 (0.14, 0.21)	0.55 (0.50, 0.60)
ADHD-BN						ADHD	BN	ADHD	BN	ADHD	BN	ADHD	BN
ACE	425.1332	30	364.7775	0.08326495	0.5166926	0.75 (0.60, 0.89)	0.22 (0, 0.83)	0.03 (0, 0.10)	0.09 (0, 0.39)	.	.	0.22 (0.13, 0.30)	0.69 (0.36, 1)
ADE	427.3113	30	364.7776	0.02287455	0.9918044	0.82 (0.69, 0.94)	0.35 (0, 0.93)	.	.	0.02 (0, 0.28)	0.13 (0, 1)	0.17 (0.02, 0.31)	0.52 (0, 1)
AE	427.4115	33	361.4115	0.25991685	-	0.82 (0.79, 0.86)	0.41 (0.31, 0.52)	.	.	.	.	0.18 (0.14, 0.21)	0.59 (0.48, 0.69)

Note: ADHD: attention-deficit hyperactivity/impulsivity disorder, AN: anorexia nervosa, OED: other eating disorders, i.e., non-AN eating disorders, BN: bulimia nervosa. In the likelihood based method (Table S5), the estimation of some of the confidence intervals (CIs) of A, C, D, and E components failed due to optimization issues. To find the estimates of the CIs, we fitted the models with weighted least square and obtained CIs using the delta method (18). Although model fitting statistics were not directly comparable to the model fitting statistics in STable 5, the p-values provided the same indications that ACE, ADE, and AE models fitted the data equally well and AE model had the lowest AIC. Estimated A, C, D, and E in each model were presented with 95% CIs. The estimates were almost the same as the heritability estimates presented in the main table Table 2, suggesting the robustness of the estimates.

Table S7. P-value of the interaction between sex and ADHD PRS and AN PRS in explaining ED and ADHD symptom measures.

	ADHD PRS thresholds (SNP p-value)						
ED measures	<0.00001	<0.001	<0.01	<0.05	<0.1	<0.5	<1 (main)
EDI-2 Full Scale	0.623	0.273	0.333	0.230	0.223	0.195	0.183
Drive for Thinness	0.446	0.355	0.335	0.425	0.390	0.318	0.278
Bulimia	0.391	0.094	0.358	0.045	0.048	0.100	0.094
Body Dissatisfaction	0.945	0.571	0.495	0.498	0.510	0.385	0.394
	AN PRS thresholds (SNP p-value)						
ADHD measures	<0.00001	<0.001	<0.01	<0.05	<0.1	<0.5	<1 (main)
A-TAC Full Scale	0.024	0.251	0.151	0.060	0.410	0.197	0.236
Inattention	0.021	0.474	0.131	0.090	0.473	0.194	0.224
Impulsivity	0.085	0.179	0.298	0.089	0.464	0.320	0.368

Note: ADHD: attention-deficit hyperactivity/impulsivity disorder, ED: eating disorders, AN: anorexia nervosa, EDI-2: eating disorder inventory-2, A-TAC: the Autism-Tics, ADHD, and Other Comorbidities inventory, PRS: polygenic risk scores. PRS at p-value threshold p<1 were used as the main measures for the primary analysis. Sex difference was not statistically significant for the main measures or PRS at most p-value thresholds (sensitivity tests), except ADHD PRS at p<0.05 and p<0.1 for bulimia sub-scale and AN PRS at p<0.00001 for A-TAC full scale and inattention sub-scale.

Table S8. How ADHD PRS was associated with ED symptom measures (EDI-2) and how AN PRS was associated with ADHD symptom measures (A-TAC) in females.

	Individual [a] No. (%)	Cronbach's Alpha	Mean (SD)	Regression coefficient (95% CI)	p-Value	R-squared
ADHD PRS and ED measures						
EDI-2 Full Scale (range 1-5.8)	3037(45.0)	0.92	2.4(0.82)	0.038 (0.003,0.072)	**0.034**	0.0020
Drive for Thinness (range 1-6)	3036(45.0)	0.89	2.5(1.10)	0.043 (-0.002,0.088)	0.063	0.0015
Bulimia (range 1-5.9)	3035(44.9)	0.74	1.6(0.61)	0.015 (-0.011,0.041)	0.248	0.0006
Body Dissatisfaction (range 1-6)	3037(45.0)	0.91	3.0(1.13)	0.05 (0.004,0.097)	**0.035**	0.0019
AN PRS and ADHD measures						
ADHD-Full Scale (range 0-19)	6747(99.9)	0.95	1.4(2.46)	-0.024 (-0.083,0.035)	0.430	0.0001
Inattention (range 0-9)	6749(99.9)	0.94	0.7(1.43)	-0.012 (-0.047,0.022)	0.480	0.0001
Impulsivity/Hyperactivity (range 0-10)	6748(99.9)	0.91	0.7(1.33)	-0.012 (-0.044,0.02)	0.466	0.0001

Note: ADHD: attention-deficit hyperactivity/impulsivity disorder, ED: eating disorders, AN: anorexia nervosa, EDI-2: eating disorder inventory-2, A-TAC: the Autism-Tics, ADHD, and Other Comorbidities inventory, PRS: polygenic risk scores. ADHD PRS and AN PRS were derived based on all reference alleles after clumping (p-value threshold <1) and were standardized before analysis. a: Number (and percentage) of individuals with the outcome measures in the female study population (N=6,754 in total). We present standardized Cronbach's alpha as a measure for internal consistency within each (sub-)scale; a higher value corresponds to high internal consistency, and the acceptable value usually range between 0.70-0.95.

Table S9. How ADHD PRS was associated with ED symptom measures (EDI-2) and how AN PRS was associated with ADHD symptom measures (A-TAC) in males.

	Individual [a] No. (%)	Cronbach's Alpha	Mean (SD)	Regression coefficient (95% CI)	p-Value	R-squared
ADHD PRS and ED measures						
EDI-2 Full Scale (range 1-5.5)	2643(39.3)	0.88	1.7(0.51)	0.015 (-0.008,0.038)	0.206	0.0008
Drive for Thinness (range 1-5.7)	2638(39.3)	0.79	1.7(0.57)	0.019 (-0.007,0.045)	0.144	0.0011
Bulimia (range 1-6)	2633(39.2)	0.69	1.4(0.49)	-0.009 (-0.031,0.013)	0.420	0.0003
Body Dissatisfaction (range 1-6)	2642(39.3)	0.85	2.1(0.86)	0.031 (-0.005,0.067)	0.094	0.0012
AN PRS and ADHD measures						
ADHD-Full Scale (range 0-19)	6704(99.8)	0.96	2.3(3.21)	-0.074 (-0.158,0.01)	0.084	0.0005
Inattention (range 0-9)	6705(99.8)	0.93	1.2(1.81)	-0.045 (-0.092,0.002)	0.063	0.0006
Impulsivity/Hyperactivity (range 0-10)	6707(99.8)	0.93	1.1(1.75)	-0.03 (-0.075,0.016)	0.202	0.0003

Note: ADHD: attention-deficit hyperactivity/impulsivity disorder, ED: eating disorders, AN: anorexia nervosa, EDI-2: eating disorder inventory-2, A-TAC: the Autism-Tics, ADHD, and Other Comorbidities inventory, PRS: polygenic risk scores. ADHD PRS and AN PRS were derived based on all reference alleles after clumping (p-value threshold <1) and were standardized before analysis. a: Number (and percentage) of individuals with the outcome measures in the male study population (N=6,718 in total). We present standardized Cronbach's alpha as a measure for internal consistency within each (sub-)scale; a higher value corresponds to high internal consistency, and the acceptable value usually range between 0.70-0.95.

Figure S1. Distributions of the full- and sub-scales of ADHD and ED symptom measures in CATSS

ADHD symptom measures (A-TAC ADHD) at age 9 or 12

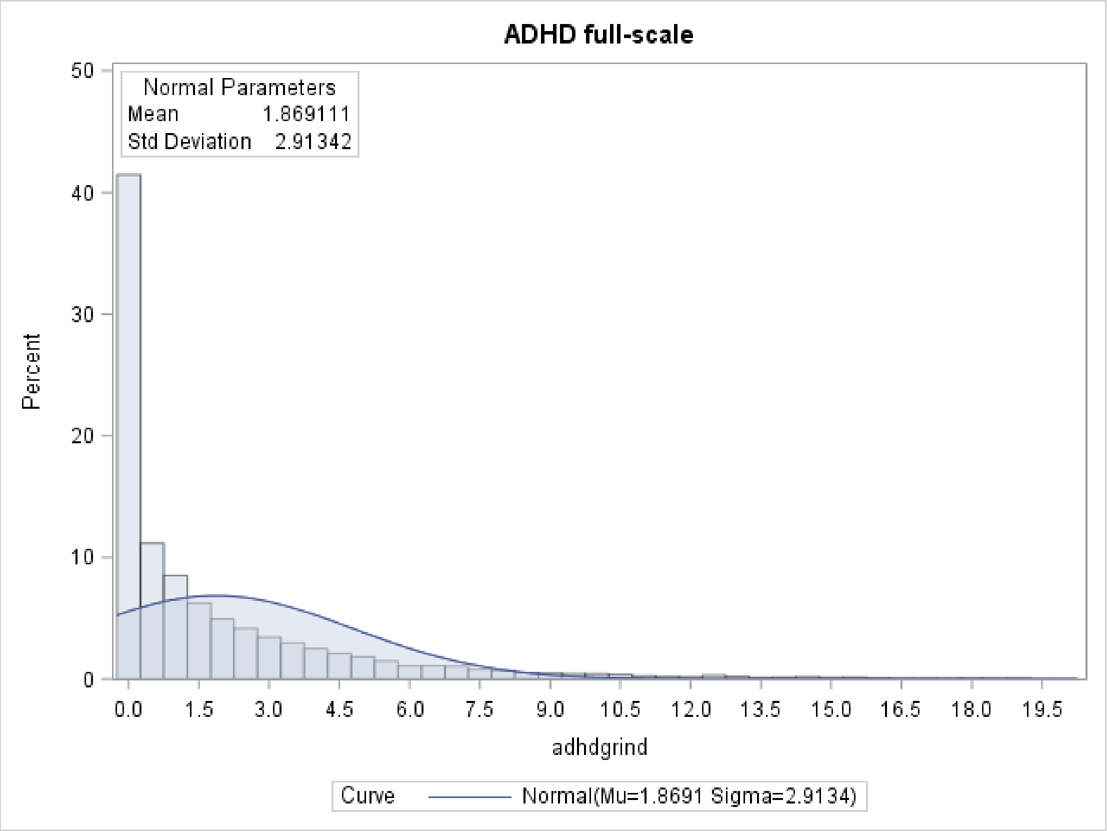

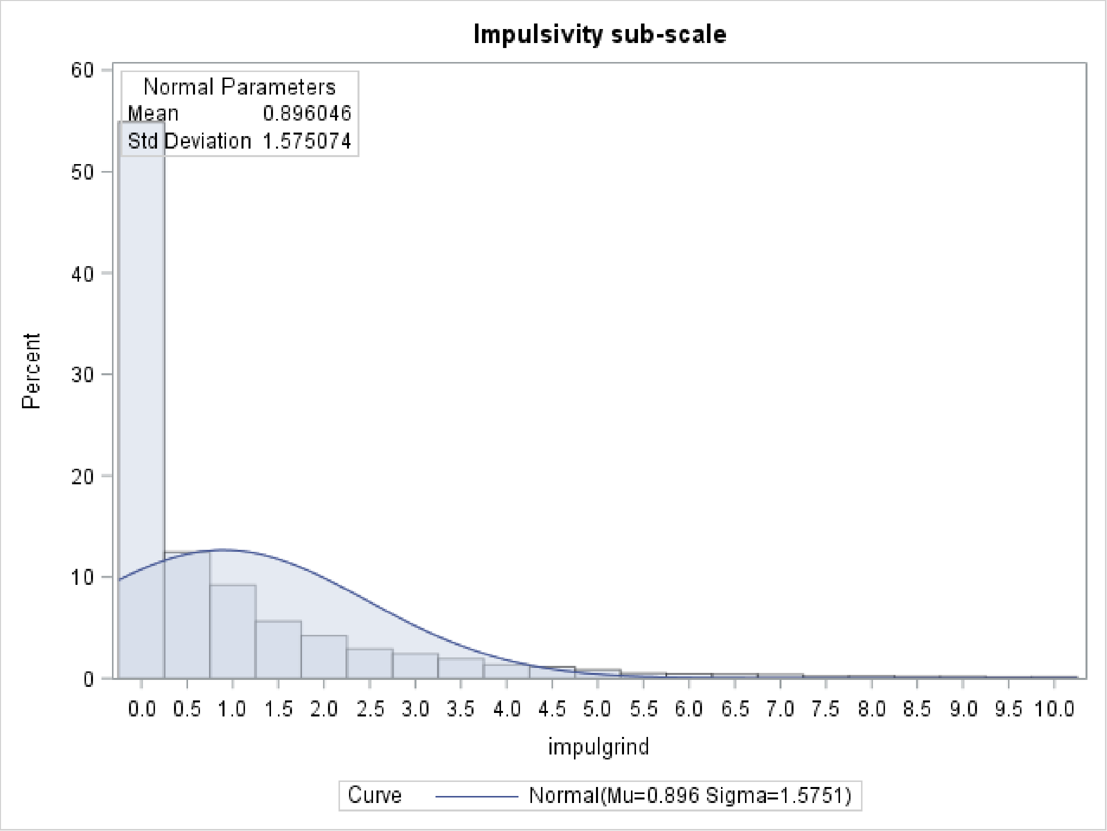

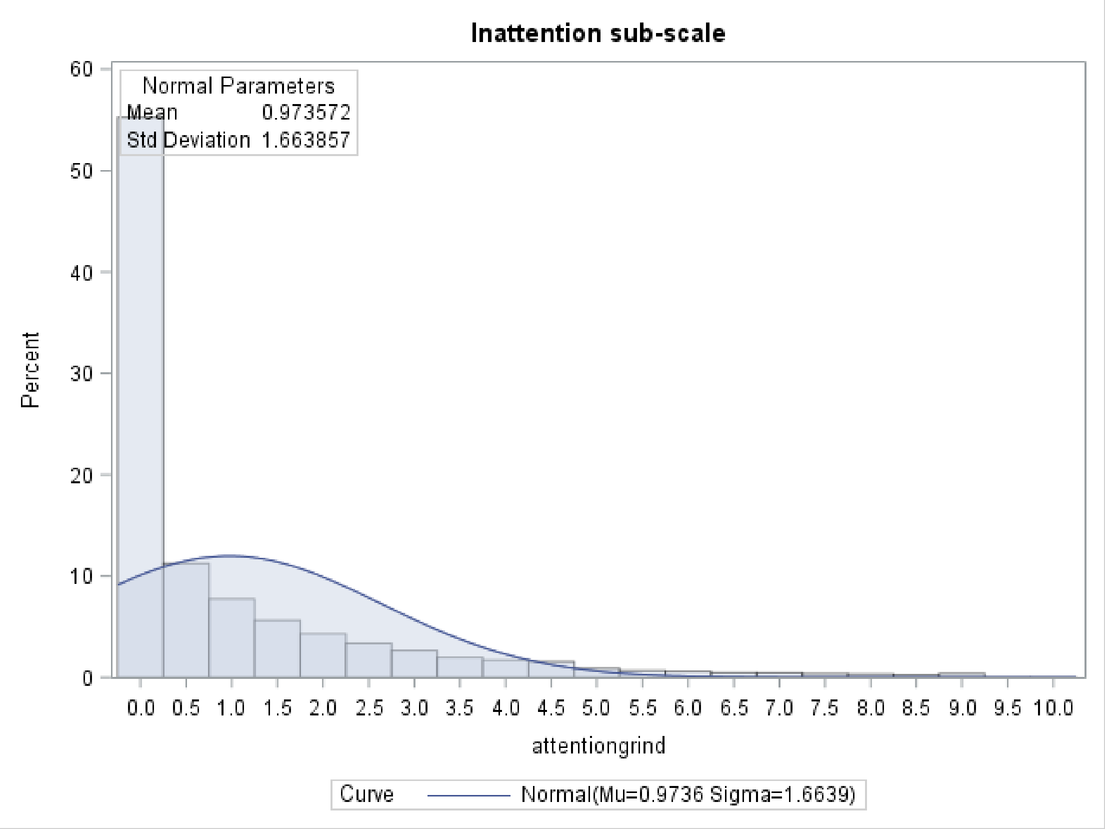

ED symptom measures (EDI-2) at age 15

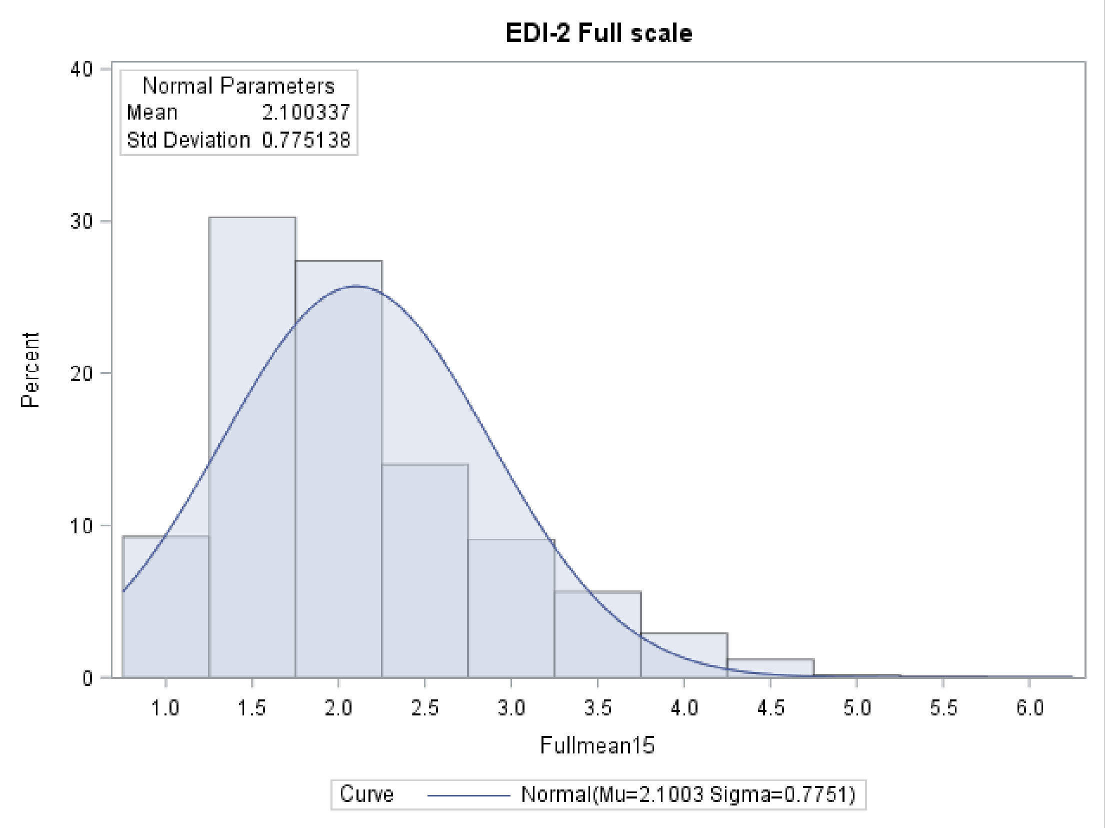

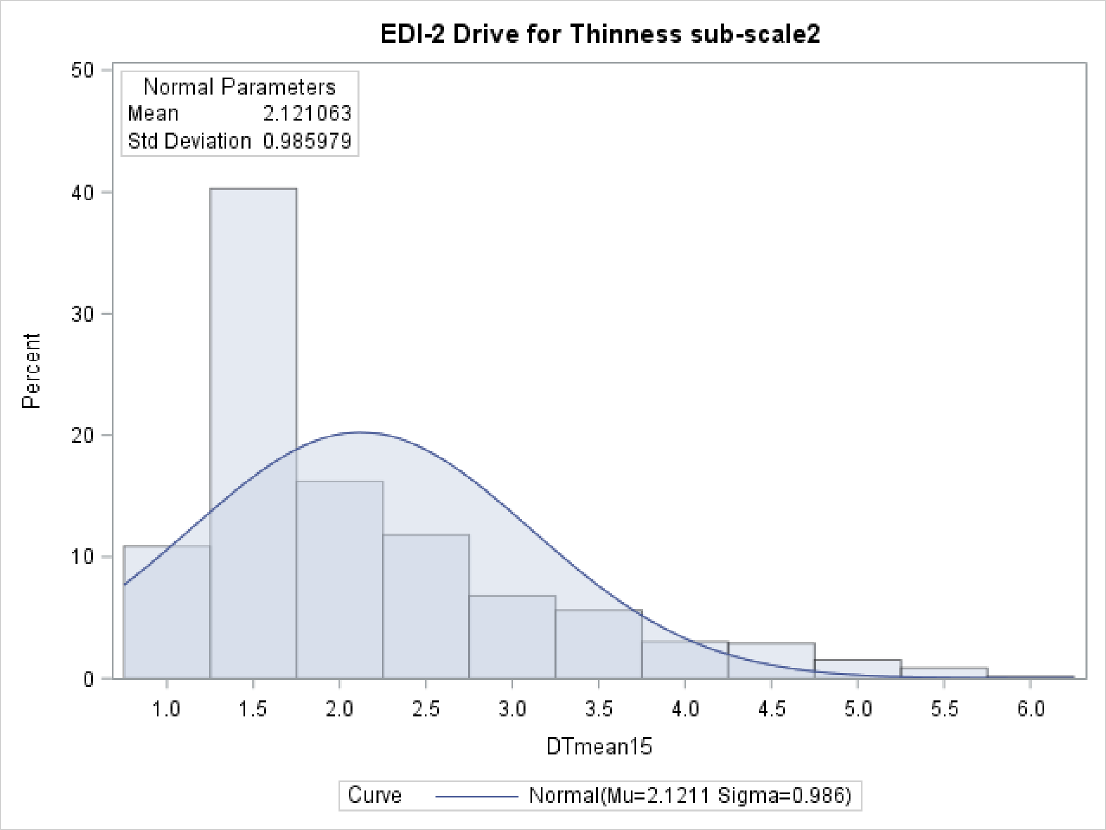
EDI-2 Drive for Thinness sub-scale2
Normal Parameters
Mean 2.121063
Std Deviation 0.985979
Percent
0
10
20
30
40
50
1.0
1.5
2.0
2.5
3.0
3.5
4.0
4.5
5.0
5.5
6.0
DTmean15
Curve
Normal(Mu=2.1211 Sigma=0.986)

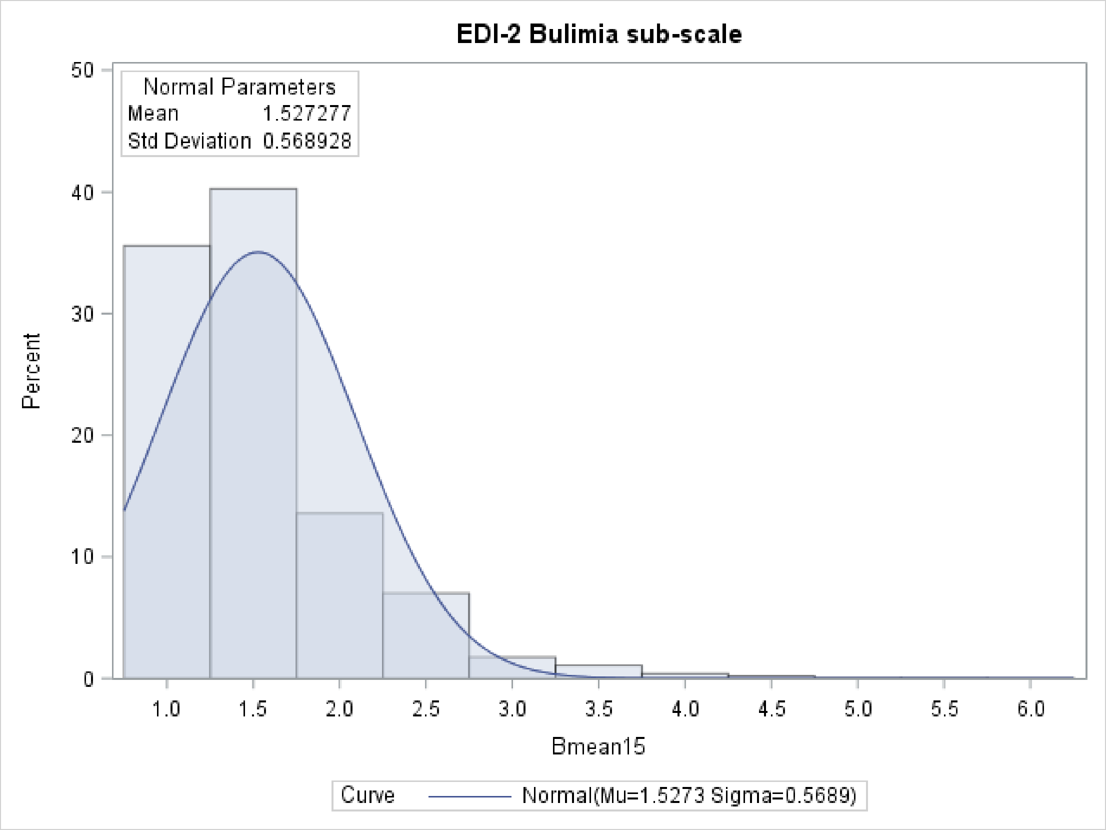
EDI-2 Bulimia sub-scale
Normal Parameters
Mean 1.527277
Std Deviation 0.568928
Percent
0
10
20
30
40
50
1.0
1.5
2.0
2.5
3.0
3.5
4.0
4.5
5.0
5.5
6.0
Bmean15
Curve
Normal(Mu=1.5273 Sigma=0.5689)

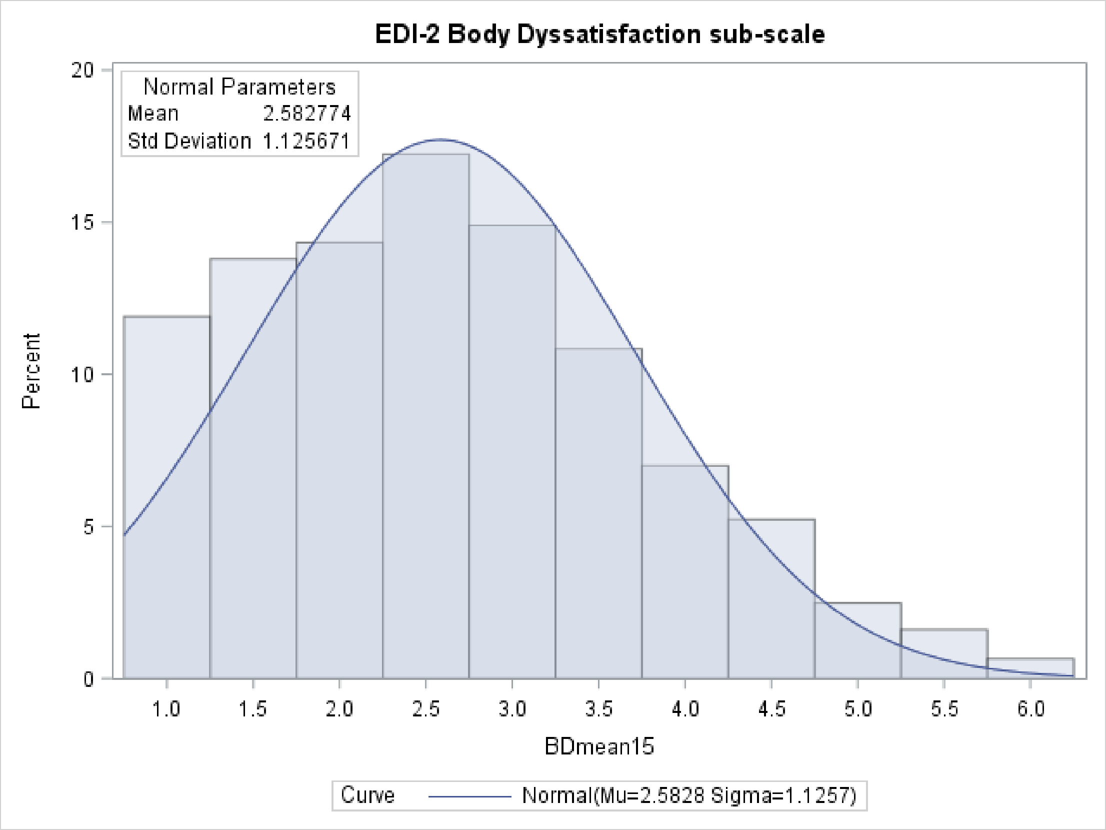

Note: ADHD: attention-deficit hyperactivity/impulsivity disorder, ED: eating disorder, A-TAC: the Autism-Tics, ADHD, and Other Comorbidities inventory, EDI-2: eating disorder inventory-2.

Figure S2. Variance explained (R-squared) and regression coefficient (beta) for the association between ADHD PRS at each p-value threshold and ED symptoms and for the association between AN PRS at each p-value threshold and ADHD symptoms in females.

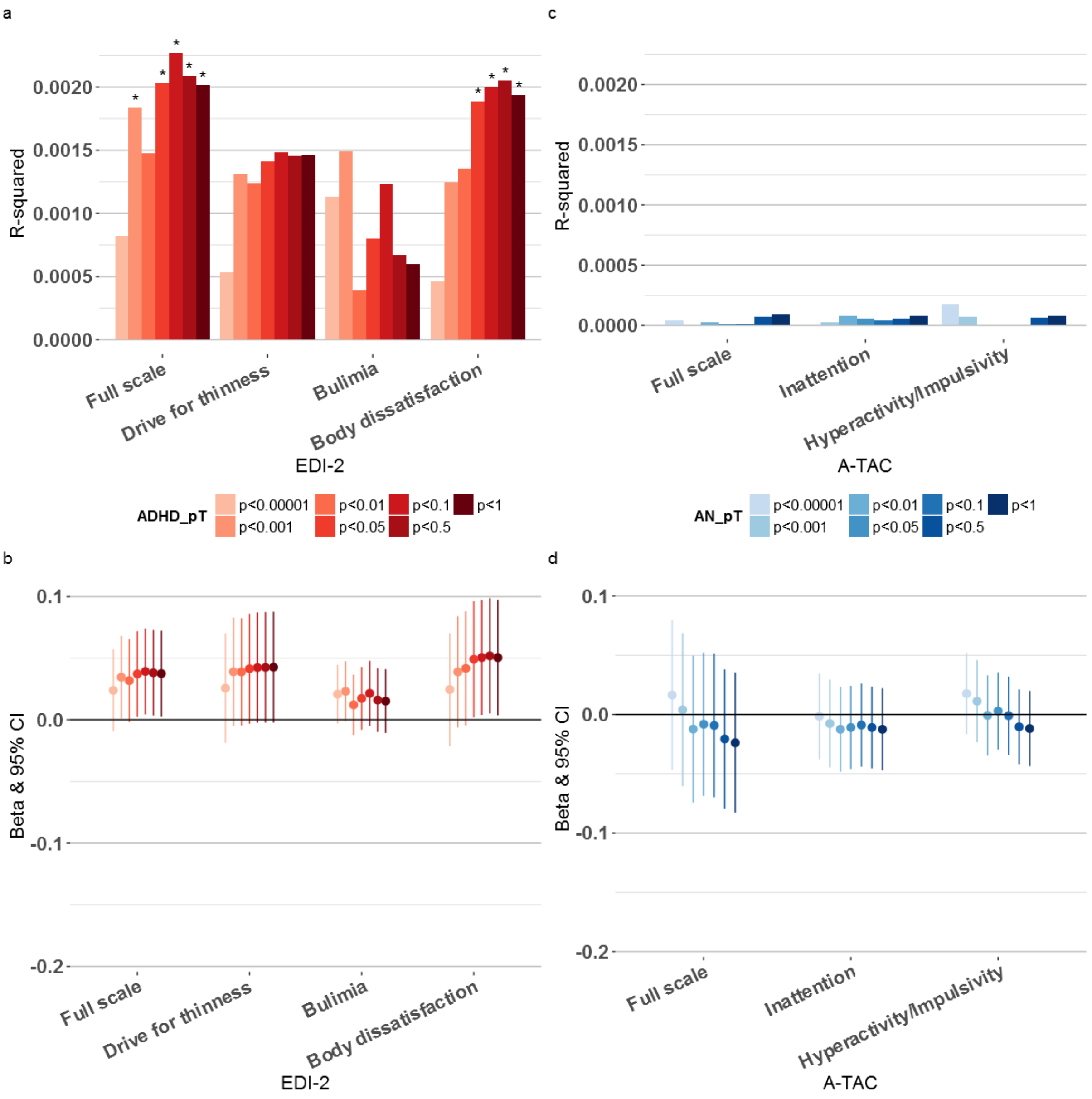

Note: ADHD: attention-deficit hyperactivity/impulsivity disorder, ED: eating disorder, AN: anorexia nervosa, EDI-2: eating disorder inventory-2, A-TAC: the Autism-Tics, ADHD, and Other Comorbidities inventory, PRS: polygenic risk scores, ADHD_pT: p-value thresholds for ADHD PRS, AN_pT: p-value thresholds for AN PRS.

* indicates $p \leq 0.05$, and ** indicates $p \leq 0.01$ for the associations between the PRS and the symptom measures.

Panels a and b show the associations between ADHD PRS and EDI-2 measures of ED symptoms in females in the study population; panel a shows variance explained, and panel b shows regression coefficients. Panels c and d show the associations between AN PRS and A-TAC measures for ADHD symptoms in females in the study population; panel c shows variance explained, and panel d shows regression coefficients.

Figure S3. Variance explained (R-squared) and regression coefficient (beta) for the association between ADHD PRS at each p-value threshold and ED symptoms and for the association between AN PRS at each p-value threshold and ADHD symptoms in males.

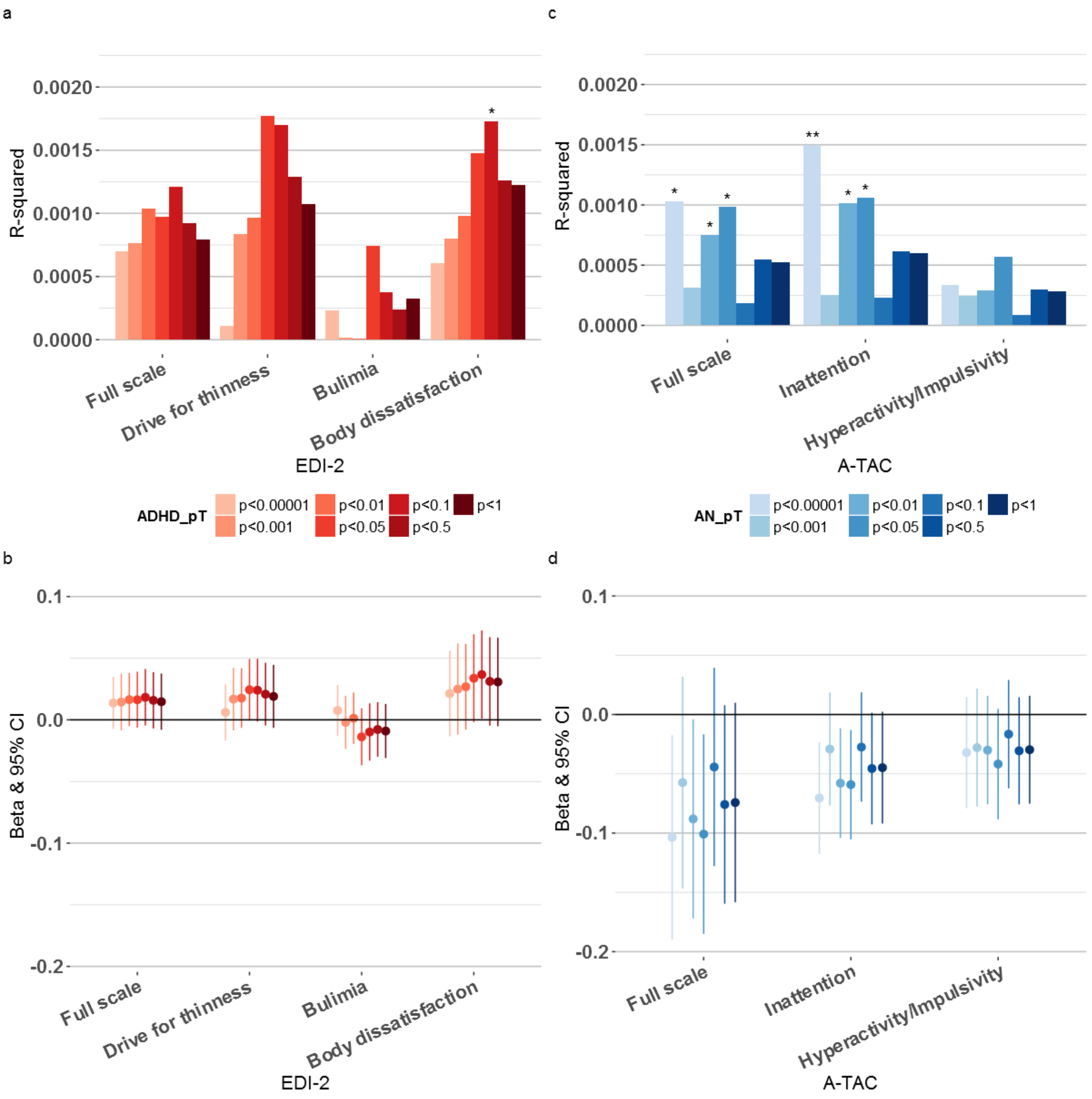

Note: ADHD: attention-deficit hyperactivity/impulsivity disorder, ED: eating disorder, AN: anorexia nervosa, EDI-2: eating disorder inventory-2, A-TAC: the Autism-Tics, ADHD, and Other Comorbidities inventory, PRS: polygenic risk scores, ADHD_pT: p-value thresholds for ADHD PRS, AN_pT: p-value thresholds for AN PRS.

* indicates $p \leq 0.05$, and ** indicates $p \leq 0.01$ for the associations between the PRS and the symptom measures.

Panels a and b show the associations between ADHD PRS and EDI-2 measures of ED symptoms in males in the study population; panel a shows variance explained, and panel b shows regression coefficients. Panels c and d show the associations between AN PRS and A-TAC measures for ADHD symptoms in males in the study population; panel c shows variance explained, and panel d shows regression coefficients.

Supplemental References

1. Ludvigsson JF, Almqvist C, Bonamy AK, Ljung R, Michaelsson K, Neovius M, et al. (2016): Registers of the Swedish total population and their use in medical research. *Eur J Epidemiol*. 31:125-136.

2. Ludvigsson JF, Andersson E, Ekbom A, Feychting M, Kim JL, Reuterwall C, et al. (2011): External review and validation of the Swedish national inpatient register. *BMC Public Health*. 11:450.

3. Javaras KN, Runfola CD, Thornton LM, Agerbo E, Birgegard A, Norring C, et al. (2015): Sex- and age-specific incidence of healthcare-register-recorded eating disorders in the complete swedish 1979-2001 birth cohort. *Int J Eat Disord*. 48:1070-1081.

4. Emilsson L, Lindahl B, Koster M, Lambe M, Ludvigsson JF (2015): Review of 103 Swedish Healthcare Quality Registries. *J Intern Med*. 277:94-136.

5. Birgegard A, Bjorck C, Clinton D (2010): Quality assurance of specialised treatment of eating disorders using large-scale Internet-based collection systems: methods, results and lessons learned from designing the Stepwise database. *Eur Eat Disord Rev*. 18:251-259.

6. APA (2000): *Diagnostic and statistical manual of mental disorders : DSM-IV-TR*. 4th ed., text revision. ed. Washington, DC: American Psychiatric Association.

7. Wettermark B, Hammar N, Fored CM, Leimanis A, Otterblad Olausson P, Bergman U, et al. (2007): The new Swedish Prescribed Drug Register--opportunities for pharmacoepidemiological research and experience from the first six months. *Pharmacoepidemiol Drug Saf*. 16:726-735.

8. Lindevall O (2009): Pastill—a comprehensive clinical database for child and adolescent psychiatry in Stockholm. *Budapest: European Society for Child and Adolescent Psychiatry*.

9. Brikell I, Larsson H, Lu Y, Pettersson E, Chen Q, Kuja-Halkola R, et al. (2017): The contribution of common genetic risk variants for ADHD to a general factor of childhood psychopathology. *bioRxiv*.

10. Das S, Forer L, Schonherr S, Sidore C, Locke AE, Kwong A, et al. (2016): Next-generation genotype imputation service and methods. *Nat Genet*. 48:1284-1287.

11. Genomes Project C, Auton A, Brooks LD, Durbin RM, Garrison EP, Kang HM, et al. (2015): A global reference for human genetic variation. *Nature*. 526:68-74.

12. Demontis D, Walters RK, Martin J, Mattheisen M, Als TD, Agerbo E, et al. (2017): Discovery Of The First Genome-Wide Significant Risk Loci For ADHD. *bioRxiv*.145581.

13. Purcell S, Neale B, Todd-Brown K, Thomas L, Ferreira MA, Bender D, et al. (2007): PLINK: a tool set for whole-genome association and population-based linkage analyses. *Am J Hum Genet*. 81:559-575.

14. Wray NR, Lee SH, Mehta D, Vinkhuyzen AA, Dudbridge F, Middeldorp CM (2014): Research review: Polygenic methods and their application to psychiatric traits. *J Child Psychol Psychiatry*. 55:1068-1087.

15. Duncan L, Yilmaz Z, Gaspar H, Walters R, Goldstein J, Anttila V, et al. (2017): Significant Locus and Metabolic Genetic Correlations Revealed in Genome-Wide Association Study of Anorexia Nervosa. *Am J Psychiatry*. 174:850-858.

16. Ware EB, Schmitz LL, Faul JD, Gard A, Mitchell C, Smith JA, et al. (2017): Heterogeneity in polygenic scores for common human traits. *bioRxiv*.

17. Yao S, Kuja-Halkola R, Thornton LM, Runfola CD, D'Onofrio BM, Almqvist C, et al. (2016): Familial Liability for Eating Disorders and Suicide Attempts: Evidence From a Population Registry in Sweden. *JAMA Psychiatry*. 73:284-291.

18. Kuja-Halkola R, D'Onofrio BM, Larsson H, Lichtenstein P (2014): Maternal smoking during pregnancy and adverse outcomes in offspring: genetic and environmental sources of covariance. *Behav Genet*. 44:456-467.
